# Structural and functional comparison of hemoglobin Glb2-1 of *Lotus japonicus* with Glb1-1 and leghemoglobins

**DOI:** 10.1093/jxb/eraf434

**Published:** 2025-09-30

**Authors:** Rosa M Esquinas-Ariza, Irene Villar, Samuel Minguillón, Ángel Zamarreño, Carmen Pérez-Rontomé, Brandon J Reeder, Niels Sandal, Deng Yan, José M García-Mina, Deqiang Duanmu, Marta Martínez-Júlvez, Manuel Becana

**Affiliations:** Departamento de Biología Vegetal, Estación Experimental de Aula Dei (EEAD), Consejo Superior de Investigaciones Científicas (CSIC), 50059 Zaragoza, Spain; Departamento de Biología Vegetal, Estación Experimental de Aula Dei (EEAD), Consejo Superior de Investigaciones Científicas (CSIC), 50059 Zaragoza, Spain; Departamento de Biología Vegetal, Estación Experimental de Aula Dei (EEAD), Consejo Superior de Investigaciones Científicas (CSIC), 50059 Zaragoza, Spain; Departamento de Biología Ambiental, Instituto Bioma, Universidad de Navarra, Pamplona 31008, Spain; Departamento de Biología Vegetal, Estación Experimental de Aula Dei (EEAD), Consejo Superior de Investigaciones Científicas (CSIC), 50059 Zaragoza, Spain; School of Life Sciences, Essex University, Colchester CO4 3SQ, UK; Department of Molecular Biology and Genetics, Aarhus University, Aarhus C 8000, Denmark; National Key Laboratory of Agricultural Microbiology, College of Life Science and Technology, Huazhong Agricultural University, Wuhan 430070, China; Departamento de Biología Ambiental, Instituto Bioma, Universidad de Navarra, Pamplona 31008, Spain; National Key Laboratory of Agricultural Microbiology, College of Life Science and Technology, Huazhong Agricultural University, Wuhan 430070, China; Departamento de Bioquímica y Biología Molecular y Celular, Facultad de Ciencias, Universidad de Zaragoza, 50009 Zaragoza, Spain; Instituto de Biocomputación y Física de Sistemas Complejos (BIFI) and GBsC Associated Unit to CSIC through EEAD, Universidad de Zaragoza, 50018, Zaragoza, Spain; Departamento de Biología Vegetal, Estación Experimental de Aula Dei (EEAD), Consejo Superior de Investigaciones Científicas (CSIC), 50059 Zaragoza, Spain; Universidad Nacional Autónoma de México, México

**Keywords:** Heme coordination, legume-rhizobium symbiosis, *Lotus japonicus*, phytohormones, pipecolic acid, plant hemoglobins, protein structure

## Abstract

The legume *Lotus japonicus* expresses nine hemoglobins, including leghemoglobins (Lbs), class 1 phytoglobin (Glb1-1), and an unusual phytoglobin (Glb2-1). Quantitative PCR, proteomics, and plant mutant analyses indicated that Glb2-1 is mainly present in nodules without replacing Lb function, but is also in roots and photosynthetic tissues. Comparison of hormonal profiles of the knock-out mutants *glb1-1*, *glb2-1*, and *glb1-1/2-1* showed that Glb1-1 and Glb2-1 have distinct functions. The increase of salicylic acid in the leaves of *glb1-1* revealed a role of Glb1-1 in the defense response, which was corroborated by accumulation of pipecolic acid, a metabolite involved in plant immunity. In contrast, the decrease of bioactive jasmonoyl-isoleucine in *glb2-1* was consistent with a role of Glb2-1 in the plant's reproductive stage. The mutants also showed changes in cytokinins, gibberellins, and polyamines, but without clear distinctive patterns. The crystal structure of Glb2-1 was determined to 1.6 Å resolution and compared with those of soybean Lb*a* and Arabidopsis Glb1. In combination with mutant versions of Glb2-1, residues Tyr31, His64, and Cys65 were identified as critical for O_2_-binding stability. Spectral changes in heme coordination when Tyr31 is substituted for Phe highlights the importance of the residue at the B10 position for Lb and Glb function.

## Introduction

Plants contain a wide diversity of hemoglobins (reviewed by Garrocho-Villegas *et al*., 2007; Hoy and Hargrove, 2008; Hill, 2012; Becana *et al*., 2020). In leguminous plants, these include ‘symbiotic hemoglobins’ (leghemoglobins; Lbs), which are expressed at millimolar concentrations in the nodules and transport O_2_ in the cytosol of infected cells (Appleby, 1984), and ‘non-symbiotic hemoglobins’ (phytoglobins; Glbs), which are expressed at (sub)micromolar concentrations in most plant tissues and perform less defined functions. Three Glb classes may be distinguished according to their phylogeny and biochemical properties. Class 1 Glbs have an extremely high O_2_ affinity, contribute to the survival of plants under hypoxia by preserving cellular energetics, and are involved in nitric oxide (NO) homeostasis (Igamberdiev and Hill, 2004; Hill, 2012; Domingos *et al*., 2015; Gupta *et al*., 2020). They are also involved in the functioning of the legume–rhizobium symbiosis (Shimoda *et al.*, 2005; Fukudome *et al.*, 2016; Berger *et al.*, 2020). Class 2 Glbs show homology with Lbs, have moderate O_2_ affinity, and may also scavenge NO *in vivo* (Hebelstrup and Jensen, 2008; Smagghe *et al*., 2009). Class 3 Glbs share homology with the ‘truncated’ hemoglobins of prokaryotes and exhibit low O_2_ affinity (Watts *et al*., 2001; Hoy and Hargrove, 2008; Sainz *et al*., 2013). The functions of class 3 Glbs in vascular plants are unknown but in the unicellular green alga *Chlamydomonas reinhardtii* they have been associated with the modulation of NO levels and nitrate reductase activity (Sanz-Luque *et al*., 2015) and with the NO-mediated response to phosphorus deprivation (Grinko *et al*., 2021 and references therein).

Hemoglobins may be present in the ferrous (2+) or ferric (3+) form, and may be pentacoordinate (5c) or hexacoordinate (6c). In 5c hemoglobins, like Lbs, one His residue coordinates the fifth position of the Fe (‘proximal His’), leaving the heme with an open site for reversible O_2_ binding, whereas a second His (‘distal His’) is positioned to stabilize the bound ligand but without coordinating the heme Fe. In 6c hemoglobins, like class 1 and class 2 Glbs, the distal His reversibly coordinates the sixth position of the Fe (Hoy *et al*., 2007; Hoy and Hargrove, 2008). The tertiary structure of Lbs is well known and similar to mammalian myoglobin. It consists of seven α-helices named A to H (it lacks the D helix of myoglobin) in which the proximal His is the eighth residue of helix F (F8) and the distal His is the seventh residue of helix E (E7). In soybean Lb*a*, E7 is His61 and F8 is His92 (Hargrove *et al*., 1997). Another important feature in studies of hemoglobin structure and function is the specificity and affinity for physiological ligands. The 2+ form binds O_2_ and carbon monoxide (CO), whereas the 3+ form binds cyanide (CN^−^) and acetate. Also, the 2+ form has very high affinity for nitric oxide (NO) but the 3+ form has low affinity for NO (Sharma *et al*., 1987). Therefore, Lbs are only functional in the 2+ form and, indeed, it is assumed that during evolution they have been recruited for O_2_ transport from class 2 Glbs (Hoy *et al*., 2007). There are a few known exceptions, such as the Lbs of the tropical legume *Aeschynomene evenia* (Quilbé *et al*., 2021) or the symbiotic hemoglobin of the non-legume tree *Parasponia andersonii* (Sturms *et al*., 2010), which have evolved from class 1 Glbs. In all cases, however, this evolution implies a transition of the coordination state from 6c to 5c. Although elegant studies have tackled this issue (Hoy *et al*., 2007), much work is still needed to understand the structural requirements for the transport and delivery of O_2_ in plant cells, as well as for NO scavenging and other potential functions of plant hemoglobins. In this respect, the unavailability of a crystal structure of a genuine class 2 Glb is a formidable obstacle.

The model legume *Lotus japonicus* expresses nine hemoglobins: three Lbs (Lb1, Lb2, and Lb3), two class 1 Glbs (Glb1-1 and Glb1-2), two class 3 Glbs (Glb3-1 and Glb3-2), and two unusual Glbs that we tentatively designated Glb2-1 and Glb2-2 because they may have intermediate properties between Lbs and class 2 Glbs (Larrainzar *et al*., 2020; Villar *et al*., 2021). Like Lbs, Glb2-1 and Glb2-2 are predominantly expressed in nodules and are down-regulated by nitrate (Minguillón *et al*., 2024b). However, Glb2-1 and Glb2-2 are expressed at much lower levels than Lbs, contain Cys residues, and show distinctive biochemical features regarding heme coordination. Notably, Glb2-1 is predominantly 6c in the 3+ form and 5c in the 2+ form. Quite surprisingly, we previously found that a knockout mutant of Glb2-1 showed developmental and metabolic alterations in non-nodulating plants, indicating that this protein may perform non-symbiotic functions (Villar *et al*., 2021). Because of its interest, here we have pursued an in-depth study of Glb2-1. This was done by determining expression of transcripts and proteins in plant tissues, hormone profiles of knockout mutants, and protein structures. The 3D structure of Glb2-1 was resolved by X-ray crystallography, analysed by site-directed mutagenesis, and compared with the structures of 5c (Lb) and 6c (Glb1-1) hemoglobins. Also, UV-visible spectroscopy was deployed to characterize the oxidation and coordination states of the heme and the ligand binding properties of the wild-type and mutant hemoglobins. Here we report structural and functional features of Glb2-1 using a multidisciplinary approach encompassing physiological, biochemical, and genetic studies.

## Materials and methods

### Biological materials

Plants of *Lotus japonicus* ecotypes Gifu B-129 and MG-20 were used in this study. Heterozygous seeds of *glb1-1* (gene IDs: LotjaGi3g1v0504500 for Gifu and Lj3g3v3338170 for MG-20) and *glb2-1* (gene IDs: LotjaGi5g1v0253250 for Gifu and Lj5g3v1699110 for MG-20) *LORE1* insertional mutants in Gifu background were obtained from *Lotus* Base (https://lotus.au.dk/) and the *glb1-1/2-1* double mutant was obtained by crossing homozygous *glb1-1* and *glb2-1* plants. The *glb1-1* and *glb2-1* mutants bear single exonic insertions and the codes of the *LORE1* lines are 30096642 and 30015049, respectively (Villar *et al*., 2021). Homozygous lines of all three mutants were selected by genotyping (Supplementary Fig. S1) followed by quantification of transcript levels in leaves, roots, and nodules (Supplementary Fig. S2). For complementation studies, three independent lines of the MG-20 ecotype were generated that overexpress Glb2-1 in *lb123* background. This mutant lacking the three Lbs was previously described (Wang *et al*., 2019). For the overexpression construct, the coding sequences of Glb2-1 with an N-terminal Strep-tag were synthesized by GenScript (Nanjing, China) and cloned into the *Lb2* expression cassette (Wang *et al*., 2019). Homozygous seeds were selected by PCR. The hygromycin gene resistance was amplified in each T_1_ plant (forward primer: CCGCATTGGTCTTGACCAAC and reverse primer: GCTTGTCGATCGACAGATCC). Two T_2_ plants from positive T_1_ with highest expression of *Glb2-1* measured by qRT-PCR (forward primer: CTCAGCCCTTCAACTAAGAG and reverse primer: CTTTAAGCACCAGGAAATGGG) were selected and T_3_ seeds were used for the experiments. When needed, nodulated plants were produced by inoculation with *Mesorhizobium loti* MAFF303099 (1.5×10^7^ cells per root for seedlings grown in plates and 1.5×10^8^ cells per root for seedlings grown in pots) following standard procedures (Minguillón *et al*., 2024b).

### Time course and complementation experiments

For time course experiments, seedlings of Gifu and *glb2-1* were nodulated on Jensen plates without nitrogen and were grown in a controlled-environment cabinet at 23 °C/21 °C (day/night), 120–140 μmol photons m^−2^ s^−1^, and 16 h photoperiod (Villar *et al*., 2021). Plant material was flash-frozen in liquid nitrogen and stored at −80 °C until use. For complementation assays, plants of MG-20, *lb123*, and the three lines overexpressing *Glb2-1* in *lb123* background were grown on Jensen plates with 0.25 mM NH_4_NO_3_ until 35 days post-inoculation (dpi) as indicated above. Plants were then phenotyped by measuring growth parameters, and nodules were harvested for gene and protein expression analyses.

### Gene and protein expression analyses

Total RNA extraction, cDNA synthesis, and transcript quantification were performed as described (Rubio *et al.*, 2019). Normalized relative quantities were calculated using the geometric means of the reference genes indicated in the figure legends. Immunoblots were carried out on 15% SDS-PAGE gels following standard protocols. The primary antibody was generated against *L. japonicus* Glb2-1 and was affinity purified (Sainz *et al.*, 2013). The secondary antibody was goat anti-rabbit IgG conjugated with horseradish peroxidase (Sigma-Aldrich). Further details are indicated in the legends. Immunoreactive proteins were detected by chemiluminescence using the SuperSignal West Pico kit (Pierce) and images were acquired using a ChemiDoc MP Imaging System (Bio-Rad).

### Proteomics search of hemoglobins in plant tissues

Non-nodulated plants were grown on Jensen plates with 0 or 0.2 mM KNO_3_ for 10 d (photosynthetically active plants), and Glbs were identified by proteomics as follows. Cotyledons, leaves, and roots were homogenized in a medium consisting of 50 mM potassium phosphate (pH 8.0), 1% (w/v) soluble polyvinylpyrrolidone-10, 0.1% (v/v) Triton X-100, and 1 mM EDTA. Soluble extracts (25–60 μg of protein per lane) were loaded on 15% SDS-PAGE gels, stained with Coomassie Brilliant Blue R-250, and bands (*∼*10–12 kDa and *∼*15–19 kDa) were excised from the gel and subjected to trypsin digestion and proteomics analysis. Proteomics was performed at the Central Research Support Service (SCAI) of the University of Córdoba (Spain) using a nanoElute nanoflow UPLC system (Bruker) for peptide separation, coupled with a trapped ion mobility spectrometry–time of flight (TIMS-TOF Pro 2) mass spectrometer (Bruker). This was operated in ‘data independent acquisition–parallel accumulation serial fragmentation’ mode. Details of UPLC and MS conditions were previously described (Minguillón *et al.*, 2024a). The *Lotus* Base was used for protein identification.

### Determination of phytohormone profiles and pipecolic acid

Leaves and roots of non-nodulated plants (49 d) and leaves and nodules of nodulated plants (42 dpi) of the four genotypes [wild type (WT), *glb1-1*, *glb2-1*, and *glb1-1/glb2-1*] were harvested in liquid nitrogen, finely ground by using metal beads in TissueLyser II (Qiagen) and stored at −80 °C before analyses. The resulting pulverized material was *∼*100 mg of leaves or roots and *∼*50 mg of nodules.

Abscisic acid (ABA), indole-3-acetic acid (IAA), salicylic acid (SA), jasmonic acid (JA), and jasmonoyl-isoleucine (JA-Ile) were quantified by HPLC–electrospray ionization–high-resolution mass spectrometry (HPLC–ESI–HRMS). Plant material was extracted with a precooled mixture of methanol–water–formic acid containing sodium diethyldithio-carbamate and a deuterium-labeled internal standard. Chromatographic separation of hormones was performed on a reverse-phase column Synergi 4 μm Hydro-RP 80 Å, 150×2 mm (Phenomenex) with a Dionex UltiMate 3000 UHPLC system coupled to the Exploris 120 mass spectrometer (Thermo Fisher Scientific). A product ion experiment was carried out in the negative-ion mode, employing multilevel calibration curves with deuterium labeled internal standards. Details of sample extraction, chromatography, and mass spectrometry procedures were given in Olaetxea *et al.* (2024).

Cytokinins (CKs) were quantified by HPLC–ESI–HRMS following published protocols (Olaetxea *et al.*, 2024). The following CKs were quantified: *trans*-zeatin, *cis*- and *trans*-zeatin ribosides, dihydrozeatin, dihydrozeatin riboside, isopentenyl adenine, and isopentenyl adenosine. Other CKs were below quantification limits: *cis*-zeatin, benzyladenosine, and ortho-, meta-, and para-topolin and their ribosides. Pulverized material was homogenized with a precooled mix of methanol–water–formic acid. Deuterium labeled internal standards were added to the extraction medium. Chromatographic separation of CKs was performed on a reverse-phase column Tracer Excel 120 ODSA 3 μm, 200×4.6 mm (Teknokroma) with a Dionex UltiMate 3000 UHPLC system coupled to the Exploris 120 mass spectrometer (Thermo Fisher Scientific), equipped with a HESI(II) source, a quadrupole mass filter, a C-Trap, an HCD collision cell, and an Orbitrap mass analyser. A product ion experiment was carried out in the positive-ion mode, employing multilevel calibration curves with deuterium labeled internal standards. Details of sample extraction and chromatographic and mass spectrometric systems are given in Olaetxea *et al.* (2024).

The following gibberellins (GAs) were quantified: GA_1_ and GA_4_ (bioactive GAs), GA_19_ (GA_1_ precursor), GA_8_ (GA_1_ catabolite), and GA_9_ (GA_4_ precursor). Pulverized plant material was suspended in 80% methanol–1% acetic acid containing internal standards and mixed by shaking for 1 h at 4 °C. The extract was kept at −20 °C overnight and then centrifuged and the supernatant dried in a vacuum evaporator. The dry residue was dissolved in 1% acetic acid and passed through an Oasis HLB (reverse phase) column as described in Seo *et al.* (2011). The dried eluate was dissolved in 5% acetonitrile–1% acetic acid, and separated using an autosampler and reverse-phase UHPLC (Accucore RP-MS column, 2.6 µm, 2.1×100 mm; Thermo Fisher Scientific) with a 5% to 50% acetonitrile gradient containing 0.05% acetic acid, at 400 µl min^−1^ over 21 min. GAs were analysed with a Q-Exactive mass spectrometer (Orbitrap detector; Thermo Fisher Scientific) by targeted selected ion monitoring. The concentrations in the extracts were determined using embedded calibration curves and the Xcalibur 4.0 and TraceFinder 4.1 SP1 programs. The internal standards for quantification of each of the four GAs were the deuterium-labeled hormones.

Polyamines were extracted and derivatized with benzoyl chloride as described (Solé-Gil *et al*., 2019). Quantification was performed with an Orbitrap Exploris 120 mass spectrometer coupled with a Vanquish UHPLC System **(**Thermo Fisher Scientific) using reverse-phase UHPLC with an Acquity PREMIER BEH C_18_ column (1.7 µm, 2.1×150 mm) (Waters). The mobile phase consisted of 0.1% formic acid in water (phase A) and 0.1% formic acid in acetonitrile (phase B). The solvent gradient program was: 30–43% B for 13 min, 43–100% B for 2 min, 100% B for 2 min, 100–0% B for 2 min, 2 min at 0% B, returning to 30% B for 2 min, and conditioning at 30% B for 2 min. The flow rate was 0.4 ml min^−1^ and the column temperature was set at 40 °C. Ionization was performed with heated electrospray ionization (H-ESI) in negative mode. A standard curve was performed with authentic standards using hexanodiamine as internal standard. TraceFinder software (Thermo Fisher Scientific) was used for data processing.

Pipecolic acid (2-piperidinecarboxylic acid; C_6_H_11_NO_2_ relative molecular mass 129.16) was identified in the framework of a metabolomic study conducted at the Max Planck Institute of Molecular Plant Physiology (Postdam, Germany). Extraction of plant material, derivatization of metabolites, and analysis by gas chromatography time-of-flight mass spectrometry (Pegasus HT TOF-MS, LECO; St Joseph, MI, USA) were performed using published protocols (Lisec *et al*., 2006). Briefly, plant pulverized material (*∼*25–50 mg) was homogenized in a ball mill with methanol (adding ribitol as internal standard) and fractionated with chloroform, and the upper polar phase was dried and stored at −20 °C until analysis. Then, samples were derivatized with methoxyamine hydrochloride and *N*-methyl-*N*-(trimethylsilyl)trifluoroacetamide. Data processing was performed using the Xcalibur software, and metabolites were annotated according to the Golm Metabolome Database (Kopka *et al*., 2005).

### Production of recombinant Glb2-1 protein and its mutants

Codon-optimized cDNA of Glb2-1 for *Escherichia coli* expression was cloned into pET-11a vector (Novagene) and expressed with a short N-terminal Strep-tag (Supplementary Fig. S3), which allowed its purification by streptavidin-affinity chromatography. Mutants C65A, H64V, and Y31F (Supplementary Fig. S3) were generated also with Strep-tag by site-directed mutagenesis (GenScript) and mutations were confirmed by protein sequencing. The purity of all proteins exceeded 95%. Details of protein production have been published (Villar *et al*., 2021), except that in this case the soluble extracts of *E. coli* cells were loaded onto a StrepTrap XT (Cytiva, Uppsala, Sweden) and then Glb2-1 and its mutant derivatives were eluted with 50 mM biotin following the manufacturer's protocols.

### UV-visible spectra and reduction assays

The UV-visible spectra of the Glb2-1 WT protein and its mutants in the ferric (3+), deoxyferrous (2+), cyano (3+CN), carboxy (2+CO), and nitrosyl (2+NO) forms were obtained using 0.1 cm cuvettes (250 µl) and a Lambda 25 spectrophotometer as previously described (Villar *et al*., 2020). The oxyferrous (2+O_2_) forms of WT and C65A proteins were obtained by reduction of the 3+ forms with 20 µM riboflavin and 1 mM NADH. The 2+O_2_ form of Y31F was obtained by elution from the streptavidin column and subsequent removal of biotin with a NAP-5 mini-column (GE Healthcare). Hemoglobin concentration was calculated using an extinction coefficient of 150 mM^−1^ cm^−1^ at 410 nm for the 3+ form. The time course for the reduction of the 3+ form of the WT and the mutant proteins was assayed in a medium containing 50 mM potassium phosphate (pH 7.0), 14–19 µM hemoglobin, 20 µM riboflavin, and 1 mM NADH to initiate the reaction (Becana *et al*., 1991). Time zero was taken immediately after NADH addition and the reaction was followed by scanning between 350 nm and 650 nm for 15 min at 25 °C.

### Crystallization, data collection, and model building

For the resolution of the crystal structure of *L. japonicus* Glb2-1, suitable crystals were produced by testing initially the conditions of the Jena Bioscience Classic, Basic, Morpheus, JCSG, and PEG_SALT screens using the hanging drop vapor diffusion method at 18 °C. Good quality crystals were obtained in drops of 1 µl of 2.2 mM protein in 20 mM Tris–HCl buffer (pH 7.0) and 1 µl of a precipitating solution containing 28% PEG 4000, 200 mM ammonium citrate, 4% polypropylene glycol, and 100 mM KCN (pH 7), equilibrated against 0.5 ml of reservoir solution. Red crystals of size 50×50×50 µm appeared after 1 week and then were soaked in a cryoprotectant solution containing 80% of mother liquor and 20% ethylene glycol. X-ray diffraction data were collected from a single crystal of Glb2-1 using the ALBA synchrotron (Barcelona) source on the XALOC BL13 beamline to a maximum resolution of 1.6 Å. The crystal belonged to the orthorhombic C 2 2 2_1_ space group with two molecules in the asymmetric unit, each one coordinated with a heme *b* prosthetic group and a cyanide anion (CN^−^), as well as one phosphate ion and 171 water molecules. The dataset was processed, scaled, and reduced with XDS (Kabsch, 1988) and SCALA (Evans, 2006) from the CCP4 package (Agirre *et al.*, 2023). The structure was solved by molecular replacement using the MOLREP program (Vagin and Teplyakov, 1997) from CCP4 and the structure of soybean Lb*a* (pdb 1BIN; Hargrove *et al*., 1997) as a search model. Automatic refinement was performed by Refmac5 (Murshudov *et al*., 1997) from the CCP4 package and alternating manual model building by WinCOOT (Emsley *et al*., 2010). The crystal structure of *L. japonicus* Glb2-1 has been deposited in the Protein Data Bank under accession code 9R3P. Modeled structures for Glb2-1 and soybean Lb*a* without ligands were generated with AlphaFold 3 (AF3) (Abramson *et al.*, 2024), and the PyMol program (Schrödinger and DeLano, 2020) was used for structure comparisons and image generation.

## Results and discussion

### Glb2-1 shows differential temporal expression in nodules but is also present in roots and photosynthetic organs

**Fig. 1.**
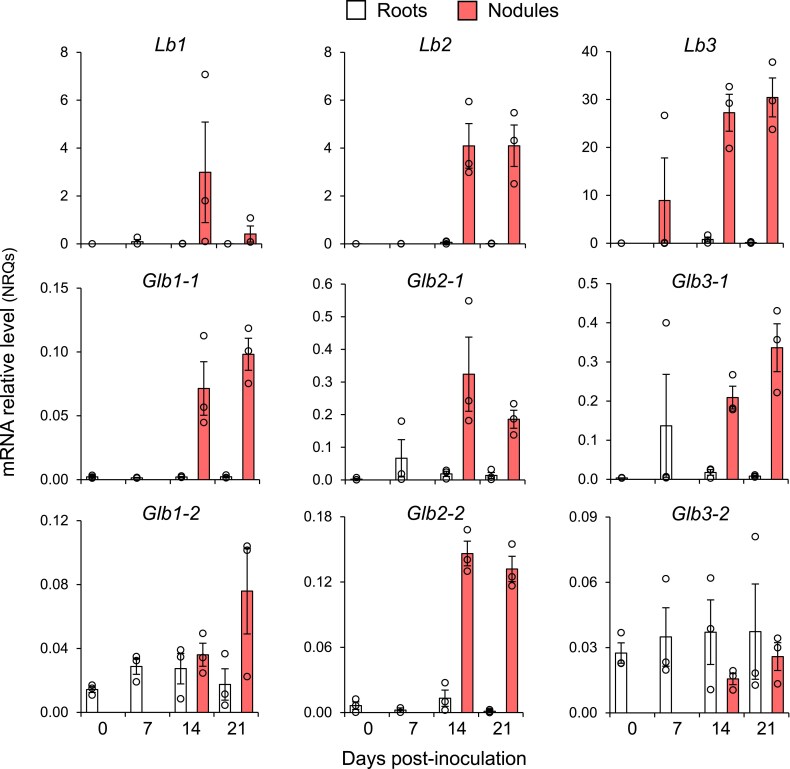
Expression of hemoglobin genes in young roots and nodules of *L. japonicus*. Roots were harvested at 0, 7, 14, and 21 days post-inoculation (dpi) and nodules at 14 and 21 dpi (when clearly visible). Transcript levels were normalized using *LjUbiquitin* and *LjATPsynthase* as the reference genes. Note that different scales were used for the individual Lbs and Glbs to facilitate meaningful visualization of expression values. Data are given in normalized relative quantities (NRQs) and are means ±SE (*n*=3). Each replicate is the pool of several plants to achieve 20–30 mg of material.

In a recent study we found that expression of *Glb2-1* in nodules of *L. japonicus* is much lower than that of *Lb* genes but decreases similarly after nitrate treatment (Minguillón *et al*., 2024b). These observations and several biochemical features of the Glb2-1 protein led us to propose that it is an evolutionary intermediate between Lbs and Glbs. To further compare these two types of hemoglobins at the early stages of nodule development, we first examined the expression of the whole set of *L. japonicus* hemoglobins between 0 and 21 dpi (Fig. 1). Roots at 7 dpi had some contaminating nodule primordia (‘bumps’), with nitrogen fixation being detectable at 10–14 dpi. Roots at 14 and 21 dpi showed fully developed nodules that were harvested separately.

As expected, expression levels of the three *Lbs* were only extremely high at 14 dpi and 21 dpi. However, *Lb1* was less expressed at 21 dpi than at 14 dpi and *Lb3* expression levels at 14 dpi were higher than those of *Lb1* and *Lb2*. This differential temporal regulation suggests that the three proteins are not fully redundant and supports a synergistic effect on nodule activity (Wang *et al*., 2019). The expression levels of *Glb2-1* and *Glb2-2* were higher in nodules than in roots but lower than those of the *Lb* genes. For example, according to the normalized relative quantities in nodules at 14 dpi, the expression levels of *Glb2-1* and *Glb2-2* were, respectively, 8% and 4% those of *Lb2* and 1.2% and 0.5% those of *Lb3* (note the different scales of expression levels in Fig. 1). These differences in magnitude are roughly consistent with those reported for pre-senescent and senescent nodules (Minguillón *et al*., 2024b). As for class 1 and class 3 hemoglobins, *Glb1-1* and *Glb3-1* are expressed much more abundantly in nodules than in the subtended roots. In sharp contrast, expression of *Glb1-2* and *Glb3-2* was prominent during the first 14 d of infection and, in particular, *Glb3-2* expression was even higher in roots than in nodules during the whole time course being examined (Fig. 1).

**Fig. 2.**
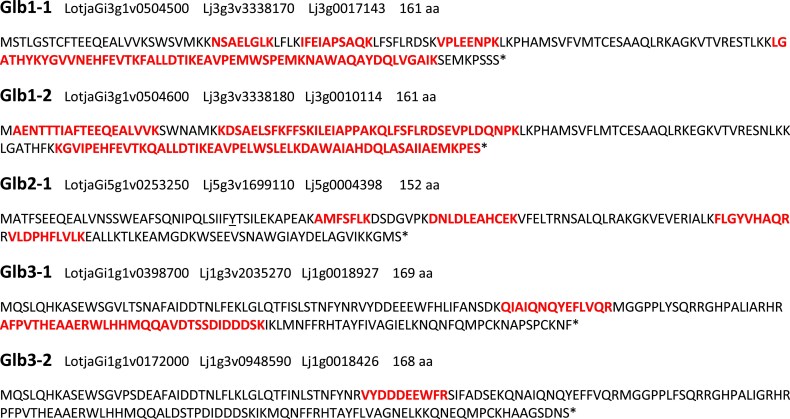
Protein sequences and tryptic peptides of phytoglobins that were detected in cotyledons, leaves, and roots of *L. japonicus* plants. Single, or in some cases adjacent, peptides are marked in red. An asterisk indicates that the C-terminus is complete. Peptides of Glb2-2 (LotjaGi5g1v0253200, Lj5g3v1699120, Lj5g0019740) were not detected. Gene accession numbers are given, sequentially, for the genomes of Gifu v1.3, MG-20 v3.0, and MG-20 version of Li *et al*. (2020). For further reference, see *Lotus* Base (https://lotus.au.dk).

Thus, our results indicate that only *Glb1-2* and *Glb3-2* are expressed at substantial levels outside of nodules. However, we previously found that the *glb2-1* mutant shows growth alterations under non-nodulating conditions (Villar *et al.*, 2021), which suggests that Glb2-1 is also functional in plant organs other than nodules. To ascertain that Glb2-1 and other Glbs, and not only their mRNAs, are expressed outside of nodules, we performed targeted proteomics in roots, leaves, and cotyledons of non-nodulated plants (Fig. 2). We could detect all Glbs except Glb2-2 in the three plant organs. This finding provides strong support for a non-symbiotic function of Glb2-1.

### Glb2-1 does not replace Lb function

To investigate if the deficiency of Glb2-1 is transcriptionally compensated for by Lbs or other Glbs, we profiled hemoglobins in nodules of the *glb2-1* mutant and found that they are similar to that of the WT nodules (Supplementary Fig. S4), suggesting that Lb does not complement Glb2-1 function. This is consistent with the apparent lack of complementation between Glb2-1 and Lbs when this was tested by using the hairy root system (Villar *et al.*, 2021). Because the functional comparison between Glb2-1 and Lb is important, here we have further studied this issue with stable transgenics, which allowed us to select high-expression lines and perform a detailed phenotypic study. To this end, *Glb2-1* was overexpressed in *lb123* background under the control of the *Lb2* promoter. Homozygous lines 6, 8, and 10 were selected based on hygromycin resistance, green fluorescent protein expression, and quantification of Glb2-1 mRNA and protein in nodules, and plants were then phenotyped (Supplementary Fig. S5). Overexpression of Glb2-1 was in the range of 13- to 25-fold at the mRNA level and was further confirmed by immunoblots. Despite these high protein levels, however, no differences were found in plant growth between the three overexpressing lines and the *lb123* mutant under symbiotic conditions, indicating that indeed this hemoglobin does not complement Lb function in nodules.

### Profiles of phytohormones and the plant defense metabolite pipecolic acid in mutants indicate that Glb1-1 and Glb2-1 have distinct functions

**Fig. 3.**
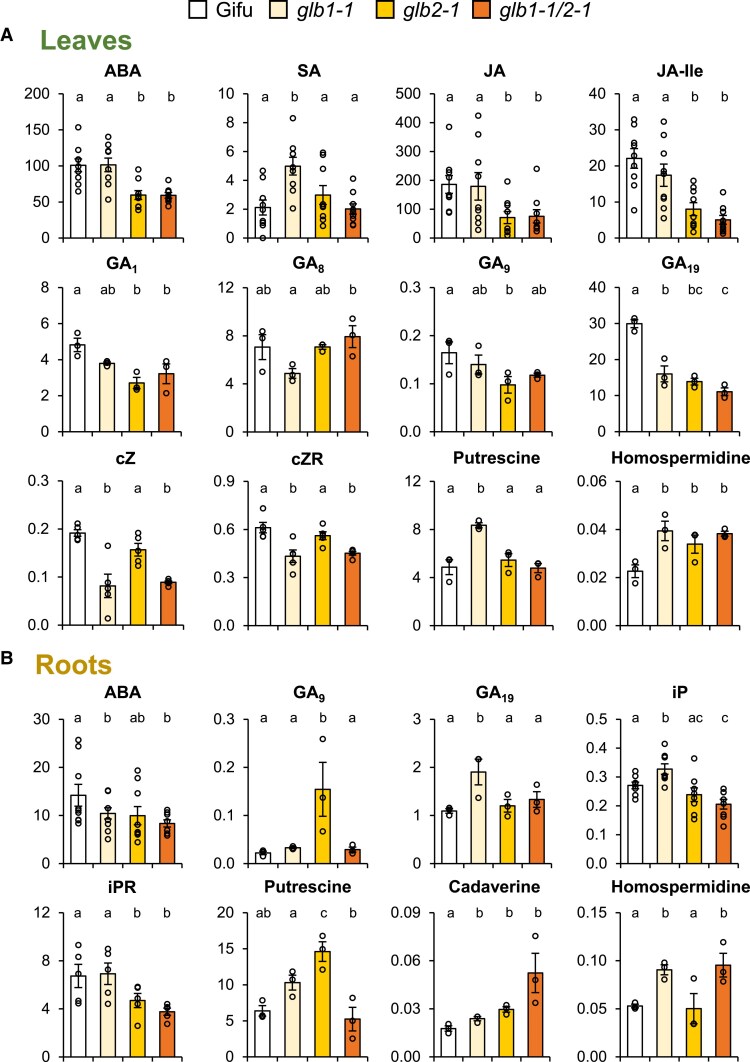
Hormonal profiles of Gifu and the *glb1-1*, *glb2-1*, and *glb1-1/2-1* mutants of *L. japonicus* plants grown under non-nodulating conditions. Only hormones with statistically significant changes in leaves (A) and roots (B) are shown. Units: acidic hormones (except SA) and CKs (pmol g^−1^ FW), SA (nmol g^−1^ FW), GAs (ng g^−1^ FW), and PAs (mg g^−1^ FW). Data are means ±SE (*n*=9 for acidic hormones, *n*=3 for GAs and PAs, *n*=for 5–9 for CKs). Different letters indicate significant differences at *P*<0.05 based on Duncan's multiple range test. ABA, abscisic acid; CK, cytokinin; cZ, *cis*-zeatin; cZR, *cis*-zeatin riboside; FW, fresh weight; GA, gibberellin; iP, isopentenyl adenine; iPR, isopentenyl adenosine; JA, jasmonic acid; JA-Ile, jasmonoyl-isoleucine; PA, polyamine; SA, salicylic acid.

**Fig. 4.**
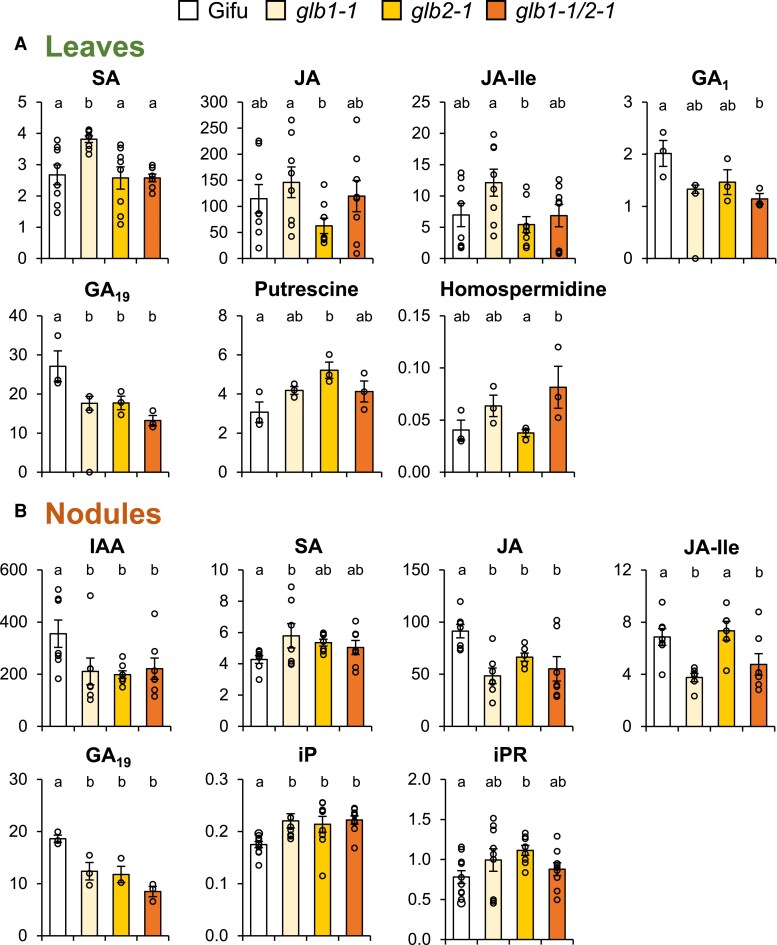
Hormonal profiles of Gifu and *glb1-1*, *glb2-1*, and *glb1-1/glb2-1* mutants of *L. japonicus* plants grown under nodulating conditions. Only hormones with statistically significant changes in leaves (A) and nodules (B) are shown. Units: acidic hormones (except SA) and CKs (pmol g^−1^ FW), SA (nmol g^−1^ FW), GAs (ng g^−1^ FW), and PAs (mg g^−1^ FW). Data are means ±SE (*n*=7–8 for acidic hormones, *n*=3 for GAs and PAs, *n*=10 for CKs). Different letters indicate significant differences at *P*<0.05 based on Duncan's multiple range test. CK, cytokinin; FW, fresh weight; GA, gibberellin; IAA, indole-3-acetic acid; iP, isopentenyl adenine; iPR, isopentenyl adenosine; JA, jasmonic acid; JA-Ile, jasmonoyl-isoleucine; PA, polyamine; SA, salicylic acid.

Next, we determined the hormone profile of *glb2-1* and compared it with those of the *glb1-1* and *glb1-1/2-1* mutants. This comparison is of interest because detailed hormonal profiles of the mutants have not been determined yet and because Glb1-1 plays a role in the regulation of NO levels in several physiological processes, including the onset of the rhizobium–legume symbiosis (Fukudome *et al*., 2016; Berger *et al*., 2020). Our previous untargeted metabolomics analysis of leaves of *glb2-1* was not sufficiently robust and only produced a few hormone identifications (Villar *et al.*, 2021). In contrast, we have used here a targeted approach to accurately quantify the acidic hormones IAA, ABA, SA, JA, JA-Ile, and GAs, as well as CKs and polyamines. This was done in leaves and roots of non-nodulated plants (Fig. 3), and in leaves and nodules of nodulated plants (Fig. 4). Among GAs, we quantified the two major bioactive forms (GA_1_ and GA_4_), their respective precursors (GA_19_ and GA_9_), and an inactive product of GA_1_ catabolism (GA_8_) (Olszewski *et al*., 2002; Binenbaum *et al*., 2018).

In both types of plants we found alterations in the contents of hormones that were specific for either *glb1-1* or *glb2-1*. In the leaves of non-nodulated plants, the contents of ABA, JA, JA-Ile, GA_1_, and GA_9_ were affected in *glb2-1* but not in *glb1-1*, whereas those of SA, *cis*-zeatin, *cis*-zeatin riboside, and putrescine changed in *glb1-1* but not in *glb2-1* (Fig. 3A). In the roots of those plants, ABA, GA_19_, isopentenyl adenine, and homospermidine were affected in *glb1-1* but not in *glb2-1*, and conversely, GA_9_, isopentenyl adenosine, and putrescine were affected in *glb2-1* but not in *glb1-1* (Fig. 3B). These results indicate that a synergistic effect of the mutations was only occasional and that Glb1-1 and Glb2-1 perform at least some independent functions. Comparison between the leaves of non-nodulated and nodulated plants (Fig. 3A versus Fig. 4A) is very interesting and reveals similarities in the patterns of SA, GA_1_, and GA_19_, but also differences in JA, JA-Ile, putrescine, and homospermidine. Furthermore, ABA and CKs were affected only in the leaves of non-nodulated plants. These observations indicate that the hormonal profiles of each mutant are also dependent on nitrogen nutrition: NH_4_NO_3_ in non-nodulated plants and NH_4_^+^ derived from N_2_ in nodulated plants. In nodules, SA and JA-Ile were affected only in *glb1-1* and isopentenyl adenosine increased significantly only in *glb2-1*, but there were also alterations shared by the two mutants, as was the case of IAA, JA, GA_19_, and isopentenyl adenine (Fig. 4B).

Relevant information may be gleaned also by examining the patterns of SA and JA in plant organs (Figs 3, 4). First, SA accumulated only in the leaves and nodules of *glb1-1*, consistent with defense or stress responses of the plant to the dysregulation of NO caused by Glb1-1 deficiency. Second, JA and/or its active form JA-Ile decreased only in the leaves and nodules of *glb2-1*. A decrease in active JA has been associated, among other physiological processes, with alterations in the reproductive stage of plants (Huang *et al*., 2017), and hence it may explain the delay in flowering, smaller pod size, and lower seed number per pod that were reported for this mutant (Villar *et al*., 2021). Third, changes in GAs and polyamines cannot be ascribed to specific mutants because they were dependent on the plant organ and nitrogen source. This can be exemplified by the variations in GA_19_, a precursor of bioactive GA_1_, or in putrescine, the most abundant polyamine by far in leaves, roots, and nodules.

**Fig. 5.**
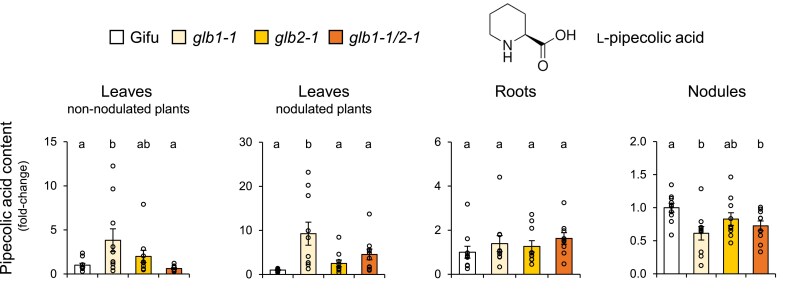
Relative contents of pipecolic acid (piperidine-2-carboxylic acid) in leaves of non-nodulated and nodulated plants, roots of non-nodulated plants, and nodules. Data are means ±SE of 10 biological replicates. Different letters indicate significant differences at *P*<0.05 based on Duncan's multiple range test.

Together, the data mentioned above indicate that Glb1-1 and Glb2-1 have non-redundant functions. To further support this, we conducted a search for primary metabolites that are differentially affected in the *glb1-1* and *glb2-1* mutants. We identified pipecolic acid as the most discriminating metabolite. This is a non-protein amino acid derived from Lys catabolism, which confers systemic acquired resistance by regulating NO and other free radicals (Wang *et al*., 2018). We found that pipecolic acid accumulates in the leaves of non-nodulated (4-fold) and nodulated (9-fold) plants of *glb1-1* but not of *glb2-1* (Fig. 5). This observation, along with the accumulation of SA in *glb1-1*, indicates that Glb1-1 is involved in the plant's defense and immune response. However, the pipecolic acid content did not change in the roots and decreased by 40% in the nodules of *glb1-1* (Fig. 5). This may be due to the fact that this metabolite is synthesized in the chloroplasts (Návarová *et al*., 2012; Wang *et al*., 2018) and hence is more abundant in the leaves (Fig. 5). Based on the decrease in JA-Ile and the absence of pipecolic acid accumulation in *glb2-1*, we also conclude that Glb2-1 is involved in a different pathway than Glb1-1, probably related to O_2_ transport (see discussion in final section).

### The crystal structure of Glb2-1 shows similarities with Lbs and differences with class 1 Glbs

**Fig. 6.**
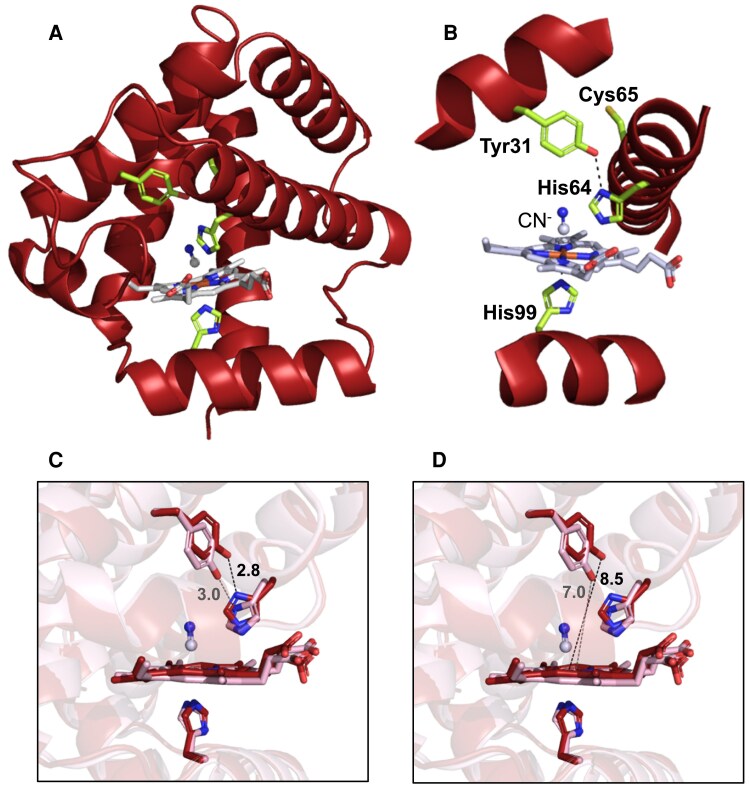
Crystal structure of *L. japonicus* Glb2-1 to 1.6 Å resolution. (A) The two key His residues (distal His64 and proximal His99) in the heme pocket, as well as Cys65, which has a potential role in O_2_ stabilization, are displayed as sticks with C atoms in light green. The heme group and CN^−^, which is coordinated with Fe^3+^ (in brown), are shown with C atoms in white and N atoms in blue. (B) CN-stabilization of Glb2-1 complex. This panel shows a close-up view of the structure in (A), with a slight rotation applied to improve visualization of all relevant residues. Note that His64 hydrogen-bonds to Tyr31 (dashed line), whereas CN^−^ occupies the sixth position of Fe coordination. (C, D) Superpositions of the cyano-3+ form (red) as obtained by the crystal structure with the 3+ form without CN^−^ (pink) as modeled by AF3, which automatically adds the heme group. Distances are indicated by dashed lines and angstrom values in the crystallographic (black) and modeled (gray) structures. Distances in (C) are for the Tyr(OH)–His coordination and distances in (D) are for the Tyr–Fe coordination. Heme groups are colored with C atoms in red and pink, respectively. Note that differences in the positions of key functional amino acid residues in the heme pocket are minor, with the main variation being the orientation of the imidazole ring of His64.

To gain further insight into the peculiarities of Glb2-1, we solved its crystal structure (Fig. 6) after repeated attempts using the 3+ form, both in the absence and presence of CN^−^ or acetate. These two ligands bind exclusively hemoglobins in the 3+ form and favor the stability of the protein for crystallization. Thus, we were only able to succesfully produce high-quality crystals when the protein was present as the cyano (3+CN) complex. Crystals diffracted to a resolution of 1.6 Å in the BL13 beamline (XALOC) of the ALBA synchrotron. The asymmetric unit contained two polypeptide molecules, each of them with one heme *b* prosthetic group and one CN^−^ along with 183 water molecules. Data collection and refinement statistics are summarized in Supplementary Table S1 and the main structural features are shown in Fig. 6. The heme pocket includes the distal His64(E7), the proximal His99(F8), and Tyr31(B10). The Cys65(E8) residue is also shown for reference of heme proximity and is the only Cys residue of Glb2-1 (Fig. 6A, B). To investigate potential structural differences in the heme cavity, we also modeled Glb2-1 in the absence of CN⁻ using AF3 (Fig. 6C, D). Whereas His99 remains unchanged in both structures, His64 and Tyr31 undergo slight positional shifts. Notably, Tyr31 retains its hydrogen bonding with His64 in the CN⁻-free model but is positioned closer to Fe^3+^ compared to the 3+CN complex.

**Fig. 7.**
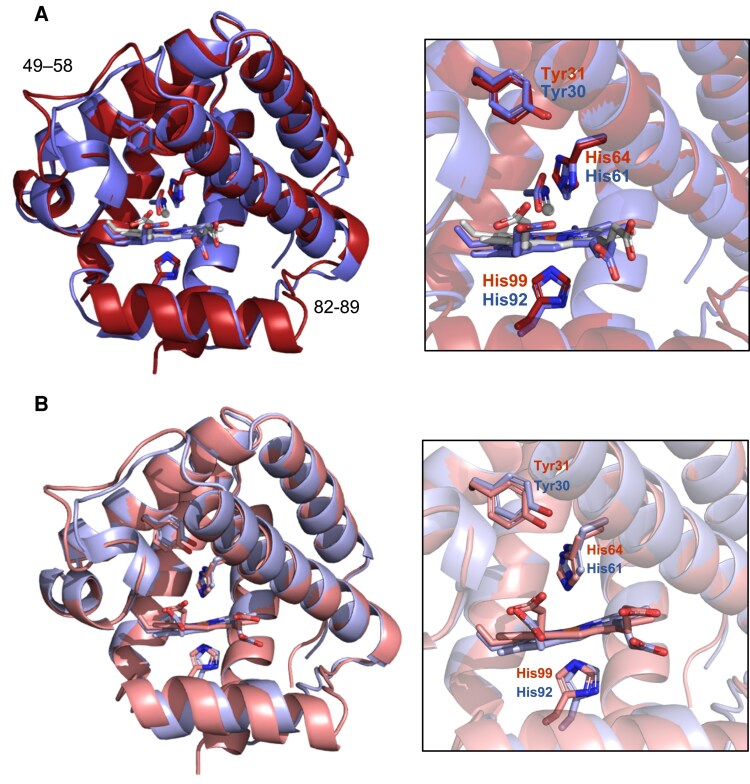
Structural comparison of Glb2-1 and soybean Lb*a* using experimental and predicted models. (A) Superposition of the crystal structure of Glb2-1 (red, pdb 9R3P) with the crystal structure of soybean Lb*a* (blue, pdb 1BIN). (B) Superposition of the AF3 cyanide-free model of Glb2-1 (light pink) with the AF3 acetate-free model of soybean Lb*a* (light blue).The right panels show close-ups of the heme cavities, with key residues displayed as sticks and numbered according to their respective sequences. The cyano group of the crystal structure of Glb2-1, the acetate molecule of the crystal structure of soybean Lb*a*, the heme groups, and the side chains of key residues are shown as sticks.

**Fig. 8.**
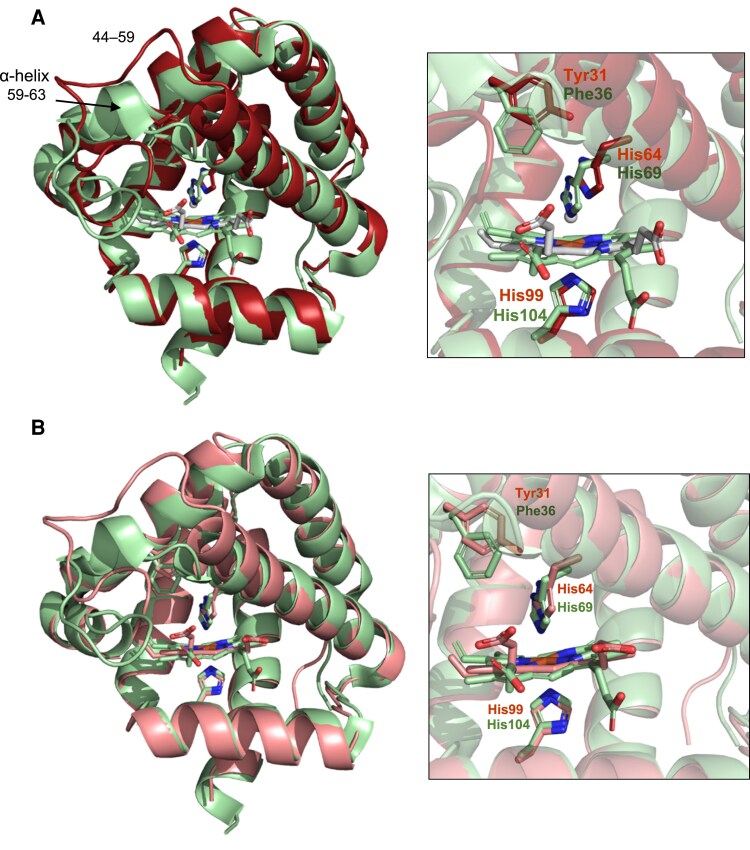
Structural comparison of Glb2-1 and AtGlb1 using experimental and predicted models. (A) Superposition of the crystal structure of Glb2-1 (red, pdb 9R3P) with the crystal structure of AtGlb1 (green, pdb 3ZHW). (B) Superposition of the AF3 cyanide-free model of Glb2-1 (light pink) with the crystal structure of AtGlb1 (green). The right panels show close-ups of the heme cavities, with key residues displayed as sticks and numbered according to their respective sequences. The cyano group of Glb2-1 in (A), the heme groups, and the side chains of key residues are shown as sticks.

To explore structural conservation, we compared the crystal structures of Glb2-1 and soybean Lb*a*, which is a typical 5c hemoglobin (Fig. 7A). The crystal structure of Glb2-1 has CN^−^ as ligand and the crystal structure of Lb contains acetate (pdb 1BIN; Hargrove *et al*., 1997). Structural superposition yielded a root mean square displacement (RMSD) of 0.57 Å for 117 Cα atoms, indicating a high degree of similarity. Differences were observed mainly in two loop regions (residues 49–58 and 82–89 in Glb2-1). We also compared the structures of Glb2-1 and Lb*a* in the absence of ligands using their respective AF3 models, observing a decrease of the distance between the distal His residues (His64 and His61, respectively) and the Fe^3+^ (Fig. 7B). A further comparison was made between Glb2-1 and a typical 6c hemoglobin, AtGlb1 (Fig. 8), which was crystallized without ligands (pdb 3ZHW; Mukhi *et al*., 2013). This comparison showed an RMSD of 2.64 Å for 149 Cα atoms, indicating slightly greater structural divergence than that observed between Glb2-1 and soybean Lb*a*. The most significant difference lies in the 44–59 loop, which in AtGlb1 (50–65) contains a short α-helix (59–63), with a RMSD of 6.38 Å for 16 Cα atoms in this region (Fig. 8A). In AtGlb1, the E7 and B10 amino acid residues (His69 and Phe36) are positioned closer to Fe^3+^ than their counterparts (His64 and Tyr31) in both the crystal structure of Glb2-1 (Fig. 8A) and its CN-free model (Fig. 8B).

It has been shown that the B10 amino acid is an important regulatory element in heme coordination in both plant (Kundu *et al*., 2004; Smagghe *et al*., 2006) and animal (Bolognesi *et al*., 1997; Pietri *et al*., 2005; Teh *et al*., 2011) hemoglobins. In particular, AtGlb1 and other class 1 Glbs with 6c coordination have a conserved Phe(B10) amino acid (Supplementary Fig. S6) that is important to stabilize O_2_ binding to Fe^2+^ (Smagghe *et al*., 2006). Thus, it is tempting to speculate that the interaction of the distal His with Phe(B10) rather than Tyr(B10) confers the extremely high O_2_ affinity to class 1 Glbs compared with Lbs. Smagghe *et al.* (2006) have pointed out for AtGlb1 that ‘in the hexacoordinate conformation, Phe(B10) interacts with His(E7) just enough to stimulate dissociation but not enough to prevent coordination’. While this is true, we know now that several Lbs do have Phe instead of Tyr at B10. This is the case, for example, of specific Lb isoforms of lupin (*Lupinus albus*), common bean (*Phaseolus vulgaris*), or cowpea (*Vigna unguiculata*) (Supplementary Fig. S6). It therefore remains to be examined how the presence of either Phe or Tyr will impact on the O_2_ binding properties of the two ‘types’ of Lbs.

### Glb2-1 mutagenesis reveals that Tyr31 is a key amino acid modulating distal His–heme coordination and that Cys65 is also involved in O_2_-binding stability

To complement the crystallographic study and get further insight into the heme environment of Glb2-1, we used UV-visible spectroscopy. The first step was to test whether the 6c coordination of Glb2-1 in the 3+ form was due to changes in ionization of an amino acid residue inside the heme cavity rather than to a *bona fide* coordination at the sixth position with an amino acid residue. To this end, we determined the spectra of Glb2-1 in the range of pH 6–12. The protein precipitated at pH 5.5 and therefore could not be tested at acidic pH. The upper panel of Supplementary Fig. S7 shows the spectrum of Glb2-1 at pH 7.0 (black trace), which is characteristic of the protein in 3+ form with 6c coordination, as shown by the Soret at 410 nm and absorption bands at 535 nm and 564 nm (shoulder, sh). Over the range tested, the optical spectrum was pH independent, indicating that there was no protonation of the distal group, consistent with a His occupying the distal position. The lower panel of Supplementary Fig. S7 shows the spectrum of Glb2-1, characteristic of the protein in 2+ form with Soret at 425 nm. At pH 7.0 (black trace), the protein is a mix of coordinations 5c at high proportion (peak at 558 nm) and 6c at low proportion (shoulder at 533 nm). In this case, when the pH was raised to 10 or 12, the protein became completely 6c, as shown by the increases of the peaks at 533 nm and 558 nm. This indicates a protonation event close to the heme pocket that results in a structural shift to form a His–Fe–His(E7) or a His–Fe–Tyr(B10) conformation.

**Fig. 9.**
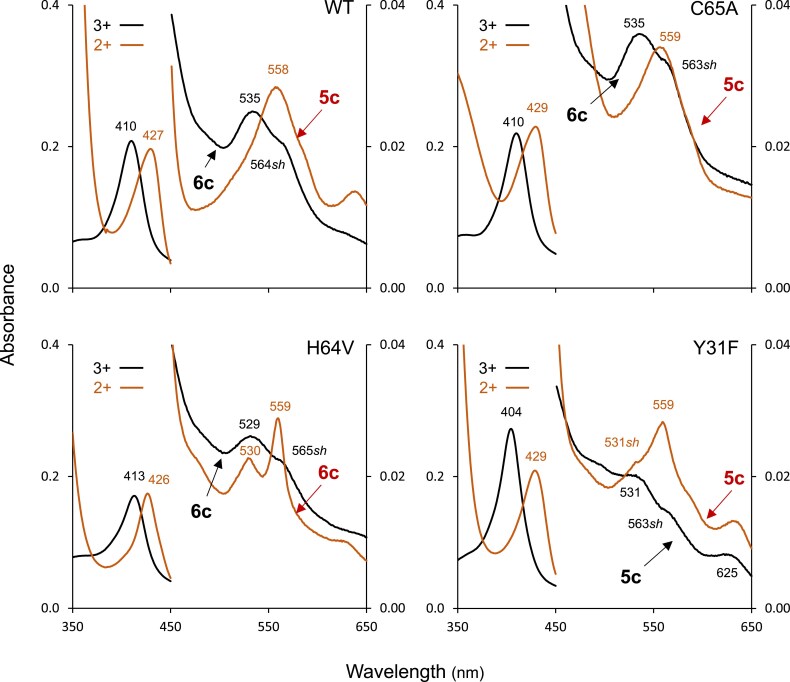
UV-visible spectra of Glb2-1 and its three mutants in the ferric (3+, black) and deoxyferrous (2+, red) forms. Protein concentrations in 50 mM potassium phosphate buffer, pH 7.0, were 17 μM (WT), 16 μM (C65A), 14 μM (H64 V), and 19 μM (Y31F). Note the following observations of the peaks and predominant coordination states (further details in the main text). (A) WT and C65A, in the 3+ form, are 6c (absorption bands at 410 nm and 535 nm and shoulder at 563–564 nm), whereas, in the 2+ form, the proteins are 5c (absorption bands at 427–429 nm and 558–559 nm). (B) H64V, in the 3+ form, is 6c (bands at 413 nm and 529 nm, and shoulder at 565 nm), and, in the 2+ form, its 6c coordination was enhanced (prominent peaks at 426, 530, and 559 nm). (C) Y31F, in the 3+ form, is 5c (bands at 404, 531 and 625 nm, with shoulder at 563 nm) and, in the 2+ form, it is mainly also 5c (429 nm and 559 nm; note that this spectrum is similar to the 2+ of WT or C65A). sh, shoulder.

The second step was to perform site-directed mutagenesis of key residues in the heme cavity, namely H64V and Y31F, and then examine the mutants by UV-visible spectroscopy. In our study we also included the C65A mutant because Cys residues are present in Glbs but not in Lbs (see positions of these three residues in Fig. 6B). All three mutant proteins are able to bind ligands of physiological interest, which include CN^−^ in the 3+ form and O_2_, CO, and NO in the 2+ form (Supplementary Fig. S8), indicating that the ligands can reach the distal heme cavity. In Fig. 9 we show the spectra of the 3+ and 2+ oxidation states of the WT and mutant proteins. Based on the comparison of the spectra, we draw the following conclusions. (i) The C65A mutation has no effect on the coordination state relative to the WT protein. In the 3+ form, the WT and C65A show characteristic absorption bands at 410 nm (Soret), 535 nm, and 563–564 nm (shoulder), typical of 6c coordination. In the 2+ form, the maxima are at 427–429 nm (Soret) and 558–559 nm, typical of 5c coordination. (ii) The coordination of the H64V mutant in the 3+ form is still 6c (same spectrum as in WT or C65A), whereas, in the 2+ form, it changed (with respect to the WT or C65A) from 5c to 6c (peaks at 530 nm and 559 nm) (Fig. 9). This result was completely unexpected and indicates that an amino acid other than His64 is responsible for the 6c coordination in the mutant protein. A plausible candidate is Tyr31, which is hydrogen bonding to His64. However, Tyr31 is not close enough to the heme as to warrant direct binding (Fig. 6D) and the effect may be indirect unless a profound conformational change takes place in the protein when His is removed. (iii) The Y31F mutation shows, also surprisingly, that the heme coordination in the 3+ form changes from 6c to 5c, as shown by a characteristic spectrum of a 5c hemoglobin, with Soret at 404 nm, absorption bands at 531 nm and 625 nm, and shoulder at 563 nm (Fig. 9). The spectrum of the 2+ form is also mainly that of a 5c hemoglobin, similar to the 2+ spectra of WT and C65A. Thus, ‘removing’ the OH group in the Y31F mutation may allow a water molecule to reach the sixth coordination position in the 3+ protein, yielding a 5c spectrum despite the presence of His64.

We conclude that the OH group of Tyr is a key player in modulating heme coordination in Glb2-1. This conclusion supports the proposal by Kundu *et al*. (2004) that Tyr30(B10) of soybean Lb*a* interacts with His61(E7) to avoid overstabilization of bound O_2_ and to allow O_2_ delivery by Lb in nodules. The same authors reported the spectra of the Y30F and H61V mutants of soybean Lb*a* (Kundu and Hargrove, 2003), and hence, it is interesting to compare them with the equivalent mutations of Glb2-1. The Soret band of Y30F of soybean Lb*a* is at 403 nm, similar to 404 nm of Y31F of Glb2-1 (Fig. 9). However, the Soret of H61V of soybean Lb*a* is at 395 nm, whereas it is at 413 nm in the H64V mutant of Glb2-1, very close to the 410 nm of the WT protein (Fig. 9). This difference also makes Glb2-1 an unsual hemoglobin that does not fit either in the Glb group, which usually contain Phe(B10), or in the Lb group, which show 5c coordination in the 3+ and 2+ forms. In a previous study we measured the O_2_ affinity of Glb2-1 (Sainz *et al*., 2013), which was found to be 11 nM, in between that of 6c Glbs (0.05–2 nM) and 5c Lbs (43 nM). Together, these results are consistent with the view that Tyr31 modulates His64 to a different extent from what it does in typical Lbs, allowing Glb2-1 to display an intermediate O_2_ affinity. This affinity is compatible with O_2_ transport (Spyrakis *et al*., 2011), whereas the extremely high O_2_ affinity of Glb1-1 is not compatible with a function in O_2_ transport and delivery (Bruno *et al*., 2007; Hoy and Hargrove, 2008).

**Fig. 10.**
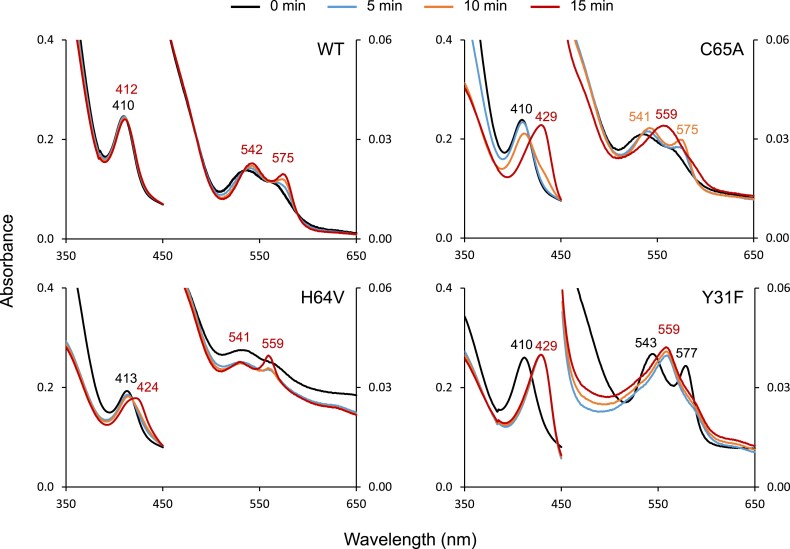
Reduction of WT and mutant versions of Glb2-1 using a riboflavin+NADH system. Note the following observations. (i) Unlike the WT protein, within 15 min, the 3+ form of C65A (6c) is reduced to the 2+deoxyferrous form (5c bands at 429 nm and 559 nm) instead of the 2+O_2_ oxyferrous form (5c bands at 541 nm and 575 nm). (ii) Reduction of the 3+ form of H64V to the 2+ form does not change the coordination state; interestingly, the protein is maintained as 6c and does not become oxygenated. (iii) Most surprisingly, the Y31F mutant becomes instantaneously reduced and oxygenated (spectrum of 2+O_2_ at time 0) and becomes quickly deoxygenated (spectra of 2+ with bands at 429 nm and 559 nm at 5, 10, and 15 min).

Finally, we performed kinetic studies for the reduction of the 3+ forms of the three mutant proteins using a riboflavin+NADH system (Fig. 10). These studies provided further useful information. Notably, the C65A mutant protein was reduced and oxygenated to the oxyferrous form (2+O_2_) at 5 min and 10 min but became deoxygenated to the 2+ form at 15 min, which indicates that Cys65 is also critical for O_2_ stabilization. Because this single Cys of Glb2-1 is not oriented towards the heme cavity (Fig. 6B), this effect may be attributed to a structural role of the Cys. In fact, Cys65 is one of the lining residues in the three main tunnels detected by CAVER 3.0 (Chovancova *et al*., 2012), when selecting His64 and Tyr31 as residues in the surroundings of the desired starting point for calculation (Supplementary Fig. S9). Such ‘tunnels’ could be the entry point of O_2_ into the heme cavity, and the presence of Cys65 in all of them may reflect a potential role of this Cys in stabilizing and/or transporting O_2_ into the heme cavity. In the case of the Y31F mutant, the protein became instantaneously reduced and oxygenated, and then deoxygenated within 5 min. Also, the H64V protein was reduced and deoxygenated within 15 min. Thus, all three mutant proteins were unable to retain bound O_2_.

### Conclusions

The hemoglobin Glb2-1 of *L. japonicus* is not a typical Lb or Glb in that it has heme coordination 6c in the 3+ form and 5c in the 2+ form, contains a Cys residue, and is a low abundance protein compared with Lbs. Here we show that Glb2-1 is expressed at the mRNA and protein levels in nodules, roots, and photosythetic tissues, where it performs symbiotic and non-symbiotic functions. However, overexpression of Glb2-1 in nodules cannot replace Lb function. Comparison of hormone profiling of the *glb1-1*, *glb2-1*, and *glb1-1/2-1* knockout mutants indicates that Glb1-1 and Glb2-1 functions may be related to SA- and JA-mediated responses, respectively. On one hand, the increase in *glb1-1* leaves of SA and pipecolic acid, a non-protein amino acid associated with the systemic acquired resistance, is consistent with a state of ‘defense response’ of the mutant in the absence of pathogens. On the other hand, the decrease of bioactive JA-Ile in *glb2-1* is consistent with alterations in the reproductive stage detected in a previous study. The crystal structure of Glb2-1, along with site-directed mutagenesis analysis, reveals that Tyr31, His64, and Cys65 are key residues for protein and O_2_-binding stabilities.

## Supplementary Material

eraf434_Supplementary_Data

## Data Availability

The data that support the findings of this study are available on request from the corresponding authors. Crystallographic data of Glb2-1 are deposited in the Protein Data Bank with entry 9R3P.

## Supplementary data

The following supplementary data are available at *JXB* online.

Fig. S1. Genotyping of *glb1-1, glb2-1,* and *glb1-1/2-1* knock-out lines of *L. japonicus*.

Fig. S2. Expression of *Glb1-1* and *Glb2-1* in knock-out lines of *L. japonicus*.

Fig. S3. Amino acid sequences of Glb2-1 (native and tagged) and of the mutant proteins, and comparison with soybean Lb*a* (GmLb*a*) and Arabidopsis Glb1 (AtGlb1).

Fig. S4. Expression profiles of the nine hemoglobins of Gifu and *glb2-1* mutant plants of *L. japonicus*.

Fig. S5. Complementation analysis of Glb2-1.

Fig. S6. Alignment of 80 sequences of Glbs and Lbs from legumes showing the B10 amino acid residues.

Fig. S7. Effect of pH on heme coordination of Glb2-1.

Fig. S8. UV-visible spectra of the complexes of Glb2-1 and its mutants with cyanide (3+CN), oxygen (2+O_2_), carbon monoxide (2+CO), and nitric oxide (2+NO).

Fig. S9. The three tunnels (surface representation) identified in the crystal structure of Glb 2-1 (red, pdb 9R3P) by CAVER 3.0, through which O_2_ could reach the heme cavity.

Table S1. X-ray diffraction data and refinement statistics for Glb2-1 of *L. japonicus*.
