# Supplementary material for: Structural and functional comparison of hemoglobin Glb2-1 of Lotus japonicus with Glb1-1 and leghemoglobins
Source: J Exp Bot. 2025 Sep 30;77(2):511–27. doi: 10.1093/jxb/eraf434 (PMC12794238; doi:10.1093/jxb/eraf434)
Supplement: eraf434_Supplementary_Data [file eraf434_supplementary_data.pdf]

Table S1. X-ray diffraction data and refinement statistics for Glb2-1 of *L. japonicus*

**Data collection statistics**	
Space group	$C222_1$
Unit cell parameters	
a, Å	35.93
b, Å	127.66
c, Å	152.73
Wavelength, Å	0.979263
Resolution, Å	152.73-1.60 (1.69-1.60)
No. of unique reflections	47061 (6768)
Redundancy	5.8 (5.9)
Completeness, %	100 (99.9)
Mn(l)/sd	15.2 (2.9)
R_{merge}^{a}	0.050 (0.434)
**Refinement Statistics**	
Resolution range, Å	76.37-1.60
Protein non-hydrogen atoms	2398
Ligand non-hydrogen atoms	95
Solvent non-hydrogen atoms	171
R_{work} (%)	17.84
R_{free}^{b} (%)	20.56
RMSD bond length, Å	0.011
RMSD bond angels, °	1.926
Mean B value (overall, A^2)	27.24

Values in parentheses correspond to the highest resolution shell.
[a] $R_{merge} = \Sigma|I - I_{av}| / \Sigma I$, where the summation is over symmetry-equivalent reflection
[b] R calculated for 7% of data excluded from the refinement.

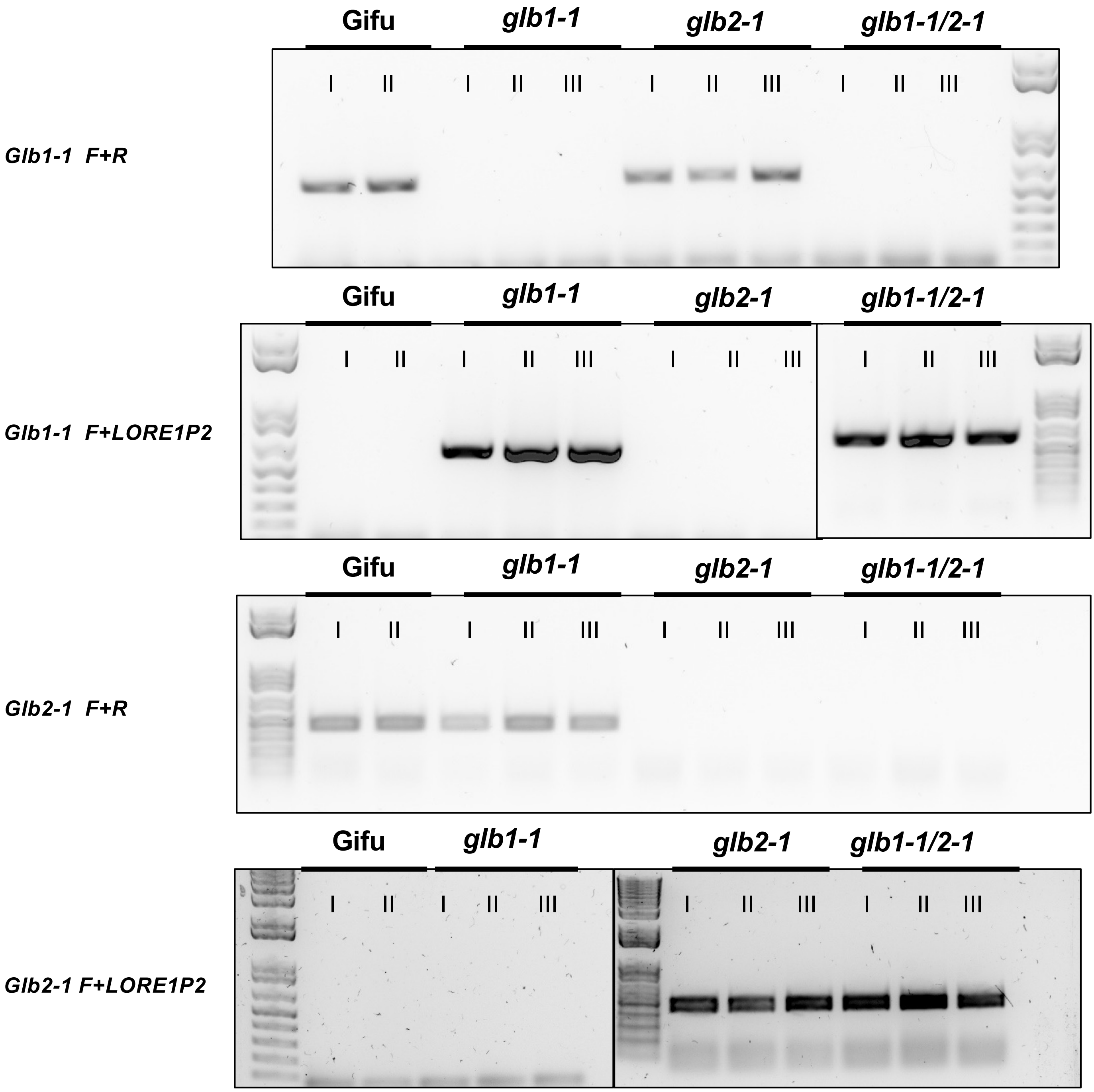

Fig. S1. Genotyping of *glb1-1, glb2-1,* and *glb1-1/2-1* knock-out lines of *L. japonicus*. Two (WT) or three (mutants) independent replicates were analyzed per genotype. The *F+R* primers amplify a DNA band exclusively from the gene locus, whereas the *F+LORE1P2* primers amplify a DNA band only from the *LORE* insert locus. Primers (5'->3') used: *Glb1-1 F* (CATGGCATGAGGCTTGAGCTTGGG), *Glb1-1 R* (TGAAACCACTCTCTTCTCGCCGCA), *Glb2-1 F* (TTCAGTGAGGAGCAAGAGGC), *Glb2-1 R* (GCCAACGGGATGGAGAAGAA), *LORE1P2* (CCATGGCGGTTCCGTGAATCT).

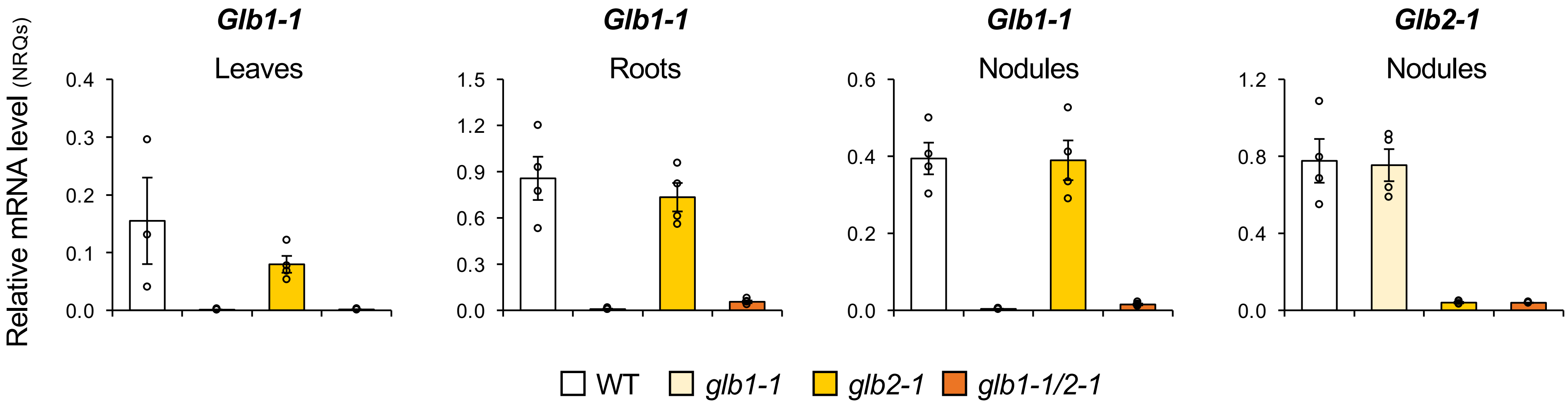

Fig. S2. Expression of *Glb1-1* and *Glb2-1* in knock-out lines of *L. japonicus*. Transcript levels were quantified in the WT and mutant plants, and are given in normalized relative quantities (NRQs). Transcript levels were normalized using *LjUbiquitin* and *LjeIF4A* as the reference genes. Measurements were made in plant organs (leaves and roots of non-nodulated plants and nodules) where the genes are mainly expressed. Data are means ± SE (*n*=3–4).

```
Glb2-1   152 aa   His64(E7, distal) His99(F8, proximal)  Tyr31(B10)  Cys65 (E8)

MATFSEEQEALVNSSWEAFSQNIPQLSIIFYTSILEKAPEAKAMFSFLKDSDGVPKDNLDLEAHCEKVFELTRNSALQ
LRAKGKVEVERIALKFLGYVHAQRRVLDPHFLVLKEALLKTLKEAMGDKWSEEVSNAWGIAYDELAGVIKKGMS*

Glb2-1 with tag   163 aa

MASWSHPQFEKGATFSEEQEALVNSSWEAFSQNIPQLSIIFYTSILEKAPEAKAMFSFLKDSDGVPKDNLDLEAHCEK
VFELTRNSALQLRAKGKVEVERIALKFLGYVHAQRRVLDPHFLVLKEALLKTLKEAMGDKWSEEVSNAWGIAYDELAG
VIKKGMS*

Glb2-1  C65A

MATFSEEQEALVNSSWEAFSQNIPQLSIIFYTSILEKAPEAKAMFSFLKDSDGVPKDNLDLEAHAEKVFELTRNSALQ
LRAKGKVEVERIALKFLGYVHAQRRVLDPHFLVLKEALLKTLKEAMGDKWSEEVSNAWGIAYDELAGVIKKGMS*

Glb2-1  H64V

MATFSEEQEALVNSSWEAFSQNIPQLSIIFYTSILEKAPEAKAMFSFLKDSDGVPKDNLDLEAVCEKVFELTRNSALQ
LRAKGKVEVERIALKFLGYVHAQRRVLDPHFLVLKEALLKTLKEAMGDKWSEEVSNAWGIAYDELAGVIKKGMS*

Glb2-1  Y31F

MATFSEEQEALVNSSWEAFSQNIPQLSIIFFTSILEKAPEAKAMFSFLKDSDGVPKDNLDLEAHCEKVFELTRNSALQ
LRAKGKVEVERIALKFLGYVHAQRRVLDPHFLVLKEALLKTLKEAMGDKWSEEVSNAWGIAYDELAGVIKKGMS*
```

```
59% identity, 74% positive

Glb2-1 1    MATFSEEQEALVNSSWEAFSQNIPQLSIIFYTSILEKAPEAKAMFSFLKDSDGVPKDNLD  60
            M  F+E+Q+ALV+SS+EAF  NIPQ S++FYTSILEKAP AK +FSFL  ++GV   N
GmLba  1    MVAFTEKQDALVSSSFEAFKANIPQYSVVFYTSILEKAPAAKDLFSFL--ANGVDPTNPK  58

Glb2-1 61   LEAHCEKVFELTRNSALQLRAKGKVEVERIALKFLGYVHAQRRVLDPHFLVLKEALLKTL  120
            L  H EK+F L R+SA QL+A G V    +A   LG VHAQ+ V DP F+V+KEALLKT+
GmLba  59   LTGHAEKLFALVRDSAGQLKASGTV----VADAALGSVHAQKAVTDPQFVVVKEALLKTI  114

Glb2-1 121  KEAMGDKWSEEVSNAWGIAYDELAGVIKKG  150
            K A+GDKWS+E+S AW +AYDELA  IKK
GmLba  115  KAAVGDKWSDELSRAWEVAYDELAAAIKKA  144


46% identity, 59% positive

Glb2-1 4    FSEEQEALVNSSWEAFSQNIPQLSIIFYTSILEKAPEAKAMFSFLKDSDGVPKDNLDLEA  63
            F+EEQEALV  SW    +N  +L +  +  I E AP  K MFSFL+DS    + N  L+
AtGlb1 9    FTEEQEALVVKSWSVMKKNSAELGLKLFIKIFEIAPTTKKMFSFLRDSPIPAEQNPKLKP  68

Glb2-1 64   HCEKVFELTRNSALQLRAKGKVEVERIALKFLGYVHAQRRVLDPHFLVLKEALLKTLKEA  123
            H   VF +   SA+QLR  GKV V    LK LG  H++  V+D HF V K ALL+T+KEA
AtGlb1 69   HAMSVFVMCCESAVQLRKTGKVTVRETTLKRLGASHSKYGVVDEHFEVAKYALLETIKEA  128

Glb2-1 124  MGDKWSEEVSNAWGIAYDELAGVIKKGMS  152
            + + WS E+  AWG AYD L   IK  M+
AtGlb1 129  VPEMWSPEMKVAWGQAYDHLVAAIKAEMN  157
```

Fig. S3. Amino acid sequences of Glb2-1 (native and tagged) and of the mutant proteins, and comparison with soybean Lb*a* (GmLb*a*) and Arabidopsis Glb1 (AtGlb1). The mutant proteins are shown untagged for simplicity. In addition to the positions of the mutated residues, the proximal histidine (His99) is also marked (in yellow) for reference. Amino acid numbering of Glb2-1 is based on the untagged protein. Asterisks at the end of the amino acid sequences mean that they are complete. Comparisons were made with Blastp (NCBI).

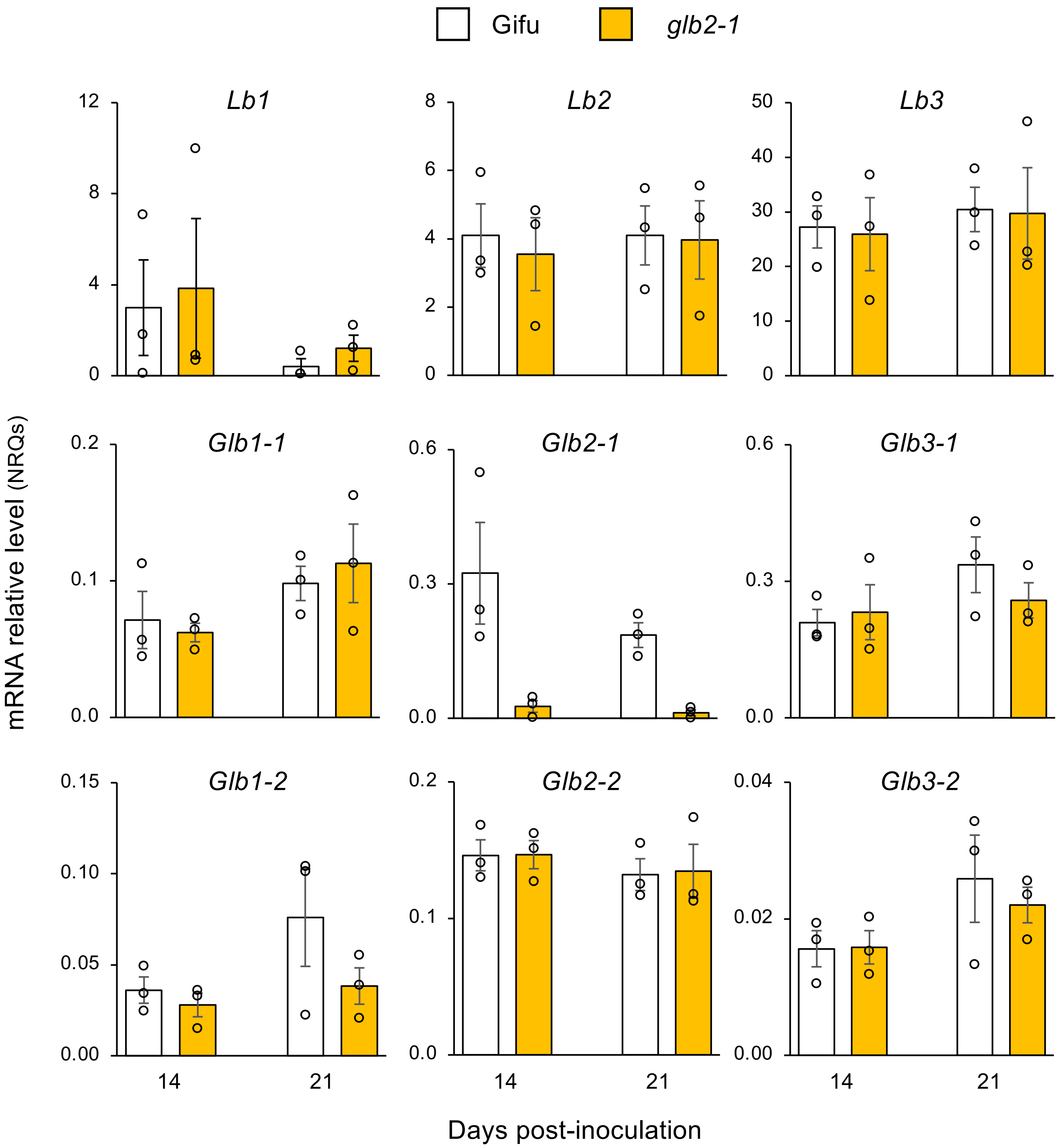

Fig. S4. Expression profiles of the nine hemoglobins in nodules of Gifu and *glb2-1* mutant plants of *L. japonicus*. Transcript levels were normalized using *LjUbiquitin* and *LjATPsynthase* as the reference genes. Data are means ± SE (*n*=3).

A Relative mRNA level (NRQ ratio of mutants vs MG-20)

MG-20	*lb123*	# 6	# 8	# 10
1.0	0.7	18.2	13.2	24.9

B

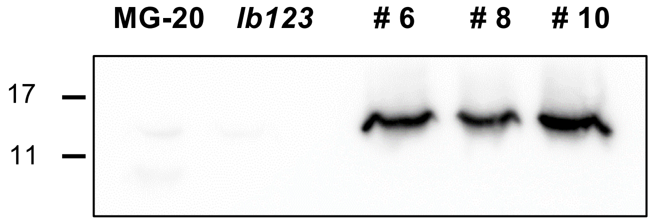

C

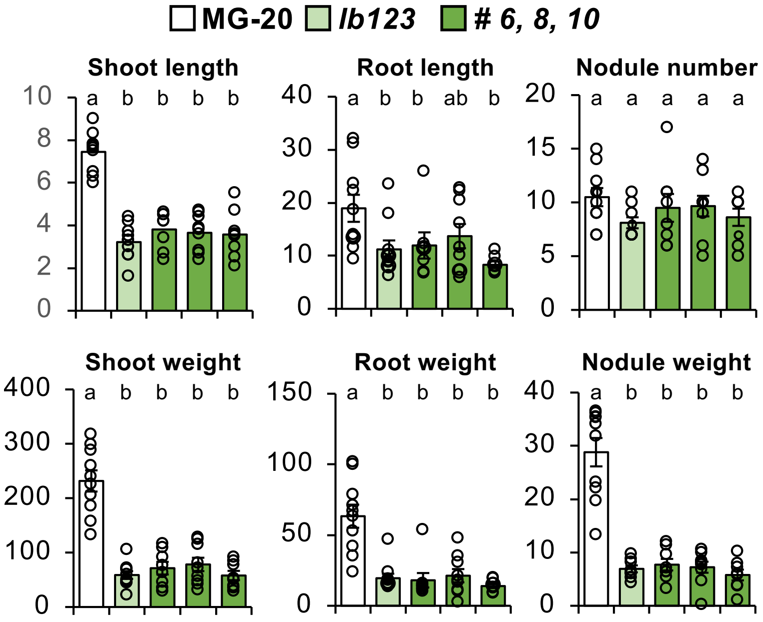

Fig. S5. Complementation analysis of *Glb2-1*. (A) Relative mRNA levels in nodules of three lines (#6, #8, and #10) overexpressing Glb2-1 in *lb123* background compared with MG-20 and *lb123* mutant. Data are means of two biological replicates with SE<15%. (B) Representative immunoblot of the same genotypes. Molecular masses (kDa) are shown on the left. Note the tenuous bands of Glb2-1 and Lbs in the WT nodules. (C) Growth parameters of the five genotypes. Lengths are expressed in cm and weights in mg. Same letters indicate no statistical differences based on Duncan's multiple range test (P<0.05). Bars represent means ± SE (n=8–10).

```
AeveLb2        ---------------MSSPLLLFSEEEEALLMNSWNVLKGNAAHLGLKFFLKI   38
AeveLb1        --------------------MAFSEEKEALVVGSWNAIKGSASDVGLKFFLKI   33
AdurLb1        --------------------MAFTAEQESLVVNSWNVLKNNSADHGLKFFLKI   33
AdurLb2        --------------------MALTAQQESLIVNSWDMLKNNSTDHGLKFFLKI   33
AdurLb3        --------------------MEFTEEQEALVVNSWDVLKNNSADLGLKFFLKI   33
CfasLb1        --------------------MGFSEQQEALVVKSWSVLKSNSEELGAKFFLKI   33
Lang1-2        ------------------MDVTFTEEQEALVMKSWNIMKKHPGDLGLKFFLKI   35
Lalb1-2        ------------------MEVTFTEEQEALVMKSWNIMKKHPGDLGLKFFLKI   35
Palb1-1        -------------MAETDGSICFTQEQEALVVKSWQVMKKNSAELGLKFFLKI   40
Mpud1-1        -------------MGESQGTICFTQEQEALVVKSWQVMKKNSAELGLKFFLKI   40
Apav1-1        --------------MSTSDGICFTEEQEALVVKSWDVMKKNSGELSLKFFLKI   39
Palb1-2        --------------MSPPEGICFTQEQEDLVVKSWDVMKKNSAELALQFFLKI   39
Mpud1-2        --------------MSTSEGICFTQEQEDLVVKSWEVMKKNSSDLALQFFLKI   39
Cfas1-1        --------------MATSEGICFTEEQEALVVKSWAAMKKNSGELGLKFFLKI   39
Eivo1          --------------MSTSEGKCFTEEQEALVVNSWGVMKKNSGELGLKFFLRI   39
Apav1-2        --------------MSASDGICFTQEQEALVVKSWEVMKKNSAELGLKFFLKI   39
Pvul1-1        ---------------MNSSGKCFTEEQEALVLKSWSVMKKNSAELGLKFFLKI   38
Mpru1          MNHTIKTLPNQATNNMSTLERGFTEEQEALVVKSWNVMKKNSGELGLKFFLKI   53
Vung1-1        ---------------MNSMGKCFTEEQEALVVKSWNVMKKNSGELGLKFFLKI   38
Vrad1-1        ---------------MNSMGKCFTEEQEALVVKSWNVMKKNSGELGLKFFLKI   38
Ccaj1-1        ---------------MTTLPRGFTEEQEALVVKSWTVMKKNSGELGLKFFLKI   38
Gmax1-1        --------------MTTTLERGFSEEQEALVVKSWNVMKKNSGELGLKFFLKI   39
Lang1-1        --------------MSTLEVRGFTEEQEALVVKSWNAMKKNSGELALKFFLKI   39
Lalb1-1        --------------MSTLEVRSFTEEQEALVVKSWNAMKKNSGELGLKFFLKI   39
Ljap1-1        --------------MSTLGSTCFTEEQEALVVKSWSVMKKNSAELGLKLFLKI   39
Aipa1          --------------MSTLEARAFTEEQEALVVKSWNVMKKNSAELGFKFFLKI   39
Adur1          --------------MSTLEATAFTEEQEALVVKSWNAMKKNSAELGFKFFLKI   39
Ovic1          ----------------MERKGGFTEEQEALVVKSWTVMKKNSAELAVKFFLKI   37
Aeve1          --------------MGSHEGREFTEEQEALVVKSWNVMKKNSAELGLKFFLKI   39
Gori1          --------------MGTLETKVFTEEQEALVVKSWAVMKKNSAELGLKFFLKI   39
Mtru1-1        --------------MGTLDTKGFTEEQEALVVKSWNAMKKNSAELGLKLFLKI   39
Tpra1-1        --------------MGTLETKGFTEEQEALVVKSWAVMKKNSAELGLKLFLKI   39
Gura1          --------------MNGLESRGFTAEQEALVVKSWNAMKKNSAELSLKFFLKI   39
Acan1          --------------MGTLERKGFTEEQEALVVKSWNAMKKNSAELSLKFFLKI   39
Olam1          --------------MDTLERKGFTEEQEALVVKSWNVMKKNSAELSLKFFLKI   39
Ljap1-2        -------------MAENTTTIAFTEEQEALVVKSWNAMKKDSAELSFKFFSKI   40
Ccaj1-2        -----------------MEARVFTEEQEALVVKSWNAIKNNSQELGLKFFNKI   36
Gmax1-2        -----------------MEGKGFTEEQEALVVKSWNEMKRNSQELGLKFFKKI   36
Vrad1-2        -----------------MDGRVFTEEQEALVVKSWAAMKKNAAEHSVKFFKKI   36
Pvul1-2        -----------------MDARVFTEEQEALVLKSWEAMKNNSGELGLKFFTKI   36
Vung1-2        -----------------MDAKVFTEEQEALVLKSWEAMKKNSGELSVKFFKKI   36
MpudLb1        -------------------MAGLSEKEVTLIKGSWEDLTKNFPEYSFYFFTQL   34
MpudLb2        -------------------MAGLSENEVTLIKGSWEDLTKKFPEYSFYFFTQL   34
LangLb3        -------------------MVAFTEKQVALVKSSWEVFNSNIPQNTHRFFTFV   34
LalbLb3        -------------------MGVFTERQVALVKSSWEVFNSNIPQNTHRFFTFV   34
LalbLb1        -------------------MAILTDVQVALVKSSFEEFNSNIPKHTHHFFTLV   34
LangLb1        -------------------MAILTDVQVALVKSSFEEFNANIPKHTHRFFTLV   34
LlutLb1        -------------------MGVLTDVQVALVKSSFEEFNANIPKNTHRFFTLV   34
LalbLb2        -------------------MGALTETQAALVKSSWEEFNANIPKHTHLFFTLV   34
LlutLb2        -------------------MGALTESQAALVKSSWEEFNANIPKHTHRFFILV   34
ApavLbY        -------------------MCGFSEREETLVRSSWEQLMQNIPYHSIRFFTSL   34
PalbLb         -------------------MGGFSEKEEALIRISWEELMKNFPHLSLRFFTLI   34
EivoLb1        -------------------MASLSENKEALVRSSWEEFMQNVPYNSCRFFTLI   34
EivoLb2        -------------------MASFGEKEEALVKSSWEEFMQNLPHNSHRFFTLI   34
EivoLb3        -------------------MASFSEKEEALVKSSWEEFTQNIPHNSRRFFTLI   34
Aeve2          -------------------MGGFTERQESLVKSSWEVLKQDIPHYSLRFFTLI   34
Lang2-1        -------------------MRAFTERQEALVKNSWEELKENIPQLSVRFFTWI   34
Lalb2-1        -------------------MRDFSERQEALVKSSWDELKQNIPQLSLRFFTWI   34
Ljap2-1        -------------------MATFSEEQEALVNSSWEAFSQNIPQLSIIFYTSI   34
MtruLb3        -------------------MDAFTERQEALVNSSWEAFKKNIPQLSILFYTLI   34
GmaxLba        -------------------MVAFTEKQDALVSSSFEAFKANIPQYSVVFYTSI   34
PvulLb1        -------------------MGAFTEKQEALVNSSWEAFKGNIPQYSVVFYTSI   34
VungLb2        -------------------MVAFSDKQEALVNGAYEAFKANIPKYSVVFYTTI   34
VradLb1        -------------------MVAFSDKQEALVNGAYEAFKADIPKYSVVFYTSI   34
PvulLb2        -------------------MGAFTEKQEALVNSSWEAFKGNLSRHSVTFFTLI   34
VungLb1        -------------------MGAFTEKQEELVNSSWEAFKGNLPQNSATFFKLV   34
VradLb2        -------------------MGGFTEKQEELVNSSWEVFKGNLSQNSVTFFKLV   34
VfabLb29       --------------------MEFTLRQEALVNSSWEAFNQNLPLFSVLFYTFI   33
LjapLb1        --------------------MGFTAQQDALVGSSYEAFKQNLPSNSVLFYTLI   33
LjapLb3        --------------------MGFTAQQEALVGSSYETFKKNLPTNSVLFYTGI   33
SrosLb2        -------------------MGAFTESQEALVTSSYETFKQNASDLSVLFYTFI   34
SrosLb1        --------------------MGFTEKQEALVNASYEAFKQNLPGNSVLFYSFI   33
OvicLb         --------------------MGYTEKQQTLVNSSWEAFKQNLPHYSVLFYTFI   33
VfabLb49       --------------------MGFTQQQEALVNSSWESFKQN-PSYSVLFYTII   32
VfabLbK        --------------------MGFTEKQEALVNSSWELFKQN-PGNSVLFYTII   32
TpraLb1        --------------------MVFTEKQEALVNGSWESFKQNLPQYSVLFYTFV   33
OspiLb         --------------------MGFTDKQEALVNSSYESFKQNLSGYSILFYTVI   33
VfabLbB        --------------------MGFTDKQEALVNSSWESFKQNLSGNSIFFYTII   33
MtruLb1        --------------------MSFTDKQEALVNSSYEAFKQNLSGYSVFFYTVI   33
TpraLb2        --------------------MGYTEKQESLVNTSWESFKQNLSGNSVLFYTII   33
```

Fig. S6. Alignment of 80 sequences of Glbs and Lbs from legumes showing the B10 amino acid residues. The Tyr(B10) and Phe(B10) residues of Lbs are highlighted in blue and yellow, respectively, and the Phe(B10) residues of Glbs are highlighted in green. Glb2-1, Lb1, and Lb3 of *L. japonicus* and soybean Lb*a* are marked in yellow.

Abbreviations for genera and species: Acan, *Astragalus canadensis*; Adur, *Arachis duranensis*; Aeve, *Aeschynomene evenia*; Aipa, *Arachis ipaensis*; Apav, *Adenanthera pavonina*; Ccaj, *Cajanus cajan*; Cfas, *Chamaecrista fasciculata*; Eivol, *Erythrophleum ivorense*; Gmax, *Glycine max*; Gori, *Galega orientalis*; Gura, *Glycyrrhiza uralensis*; Lalb, *Lupinus albus*; Lang, *Lupinus angustifolius*; Ljap, *Lotus japonicus*; Llut, *Lupinus luteus*; Mtru, *Medicago truncatula*; Mpru, *Mucuna pruriens*; Mpud, *Mimosa pudica*; Olam, *Oxytropis lambertii*; Ospi, *Ononis spinosa*; Ovic, *Onobrychis vicifolia*; Palb, *Prosopis alba*; Pvul, *Pisum vulgaris*; Sros, *Sesbania rostrata*, Tpra, *Trifolium pratense*; Vfab, *Vicia faba*, Vrad, *Vigna radiata*; Vung, *Vigna unguiculata*.

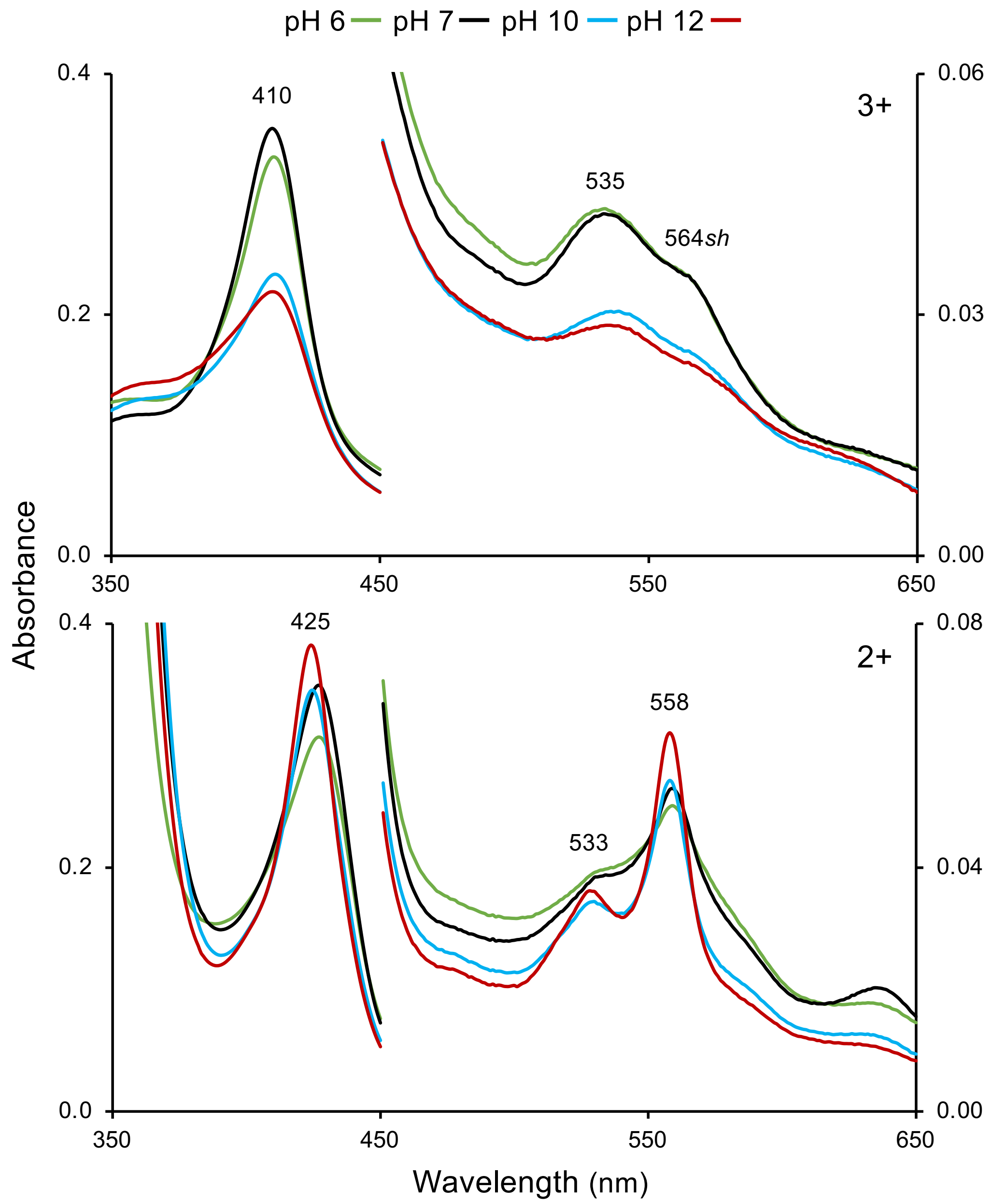

Fig. S7. Effect of pH on heme coordination of Glb2-1. The upper panel shows the spectrum of Glb2-1 at pH 7.0, characteristic of a hemoglobin in the 3+ form with coordination 6c, as indicated by the absorption bands at 410 nm (Soret), 535 nm, and 564 nm (sh, shoulder). This spectrum was not affected by alkaline pH. The lower panel shows the spectrum of Glb2-1 at pH 7.0, characteristic of the hemoglobin in 2+ form and mostly with coordination 5c, as indicated by the absorption bands at 425 and 558 nm. In this case, the proportion of 6c increased considerably at pH 10 and 12, as shown by the increasing peak at 533 nm. The buffers used were MES (pH 6), sodium phosphate (pH 7 and 8), and sodium bicarbonate (pH 10 and 12).

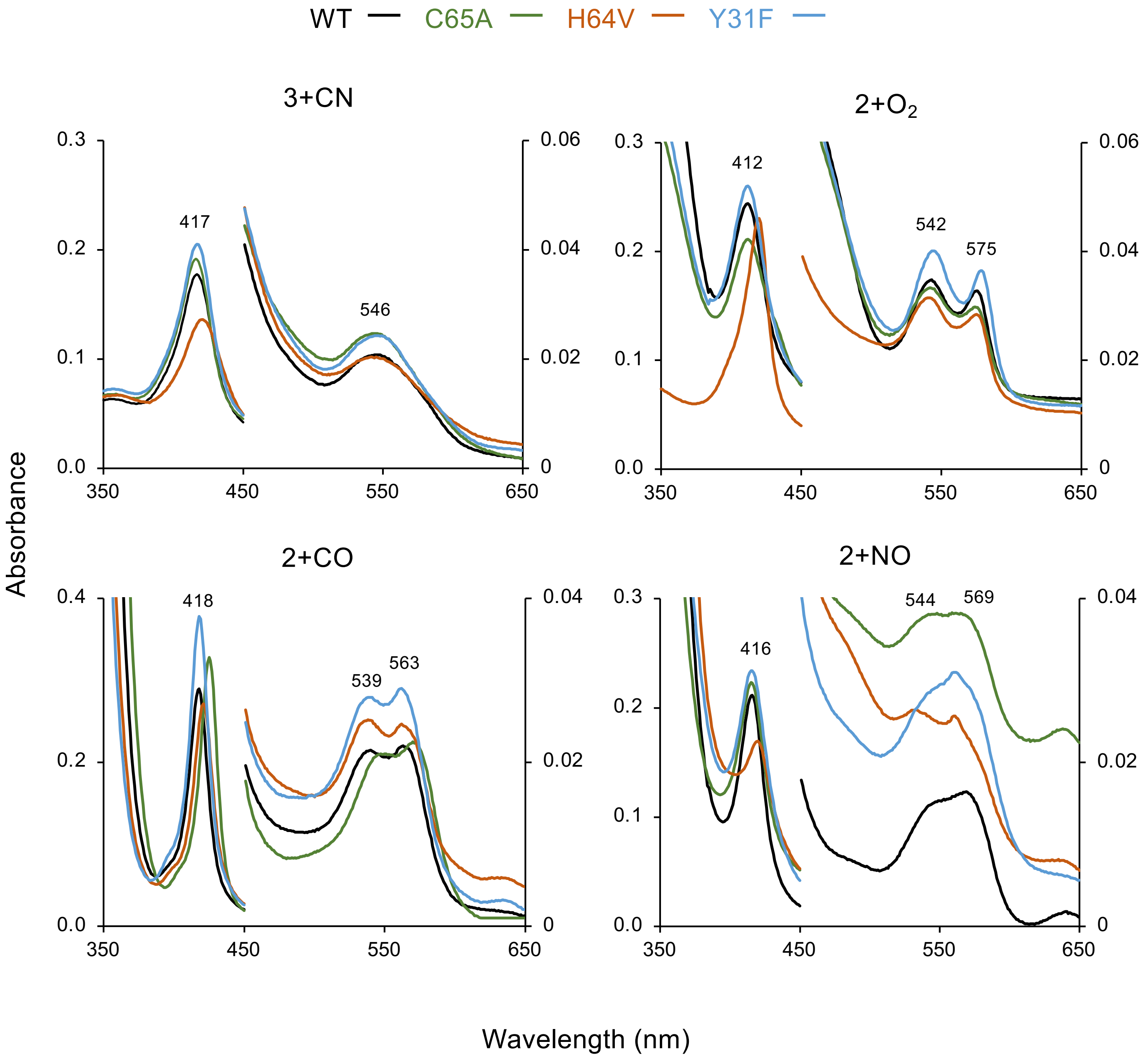

Fig. S8. UV-visible spectra of the complexes of Glb2-1 and its mutants with cyanide (3+CN), oxygen (2+O_2), carbon monoxide (2+CO), and nitric oxide (2+NO). For clarity, only some maxima of the Soret (380–430 nm) and Q absorption bands (450–650 nm) are indicated. For WT and Y31F, Soret maxima are at 417, 412, 418, and 416 nm, respectively. For C65A, Soret maxima are at 417, 419, 418, and 416 nm, respectively. For H64V, Soret maxima are at 421, 416, 421, and 419 nm, respectively.

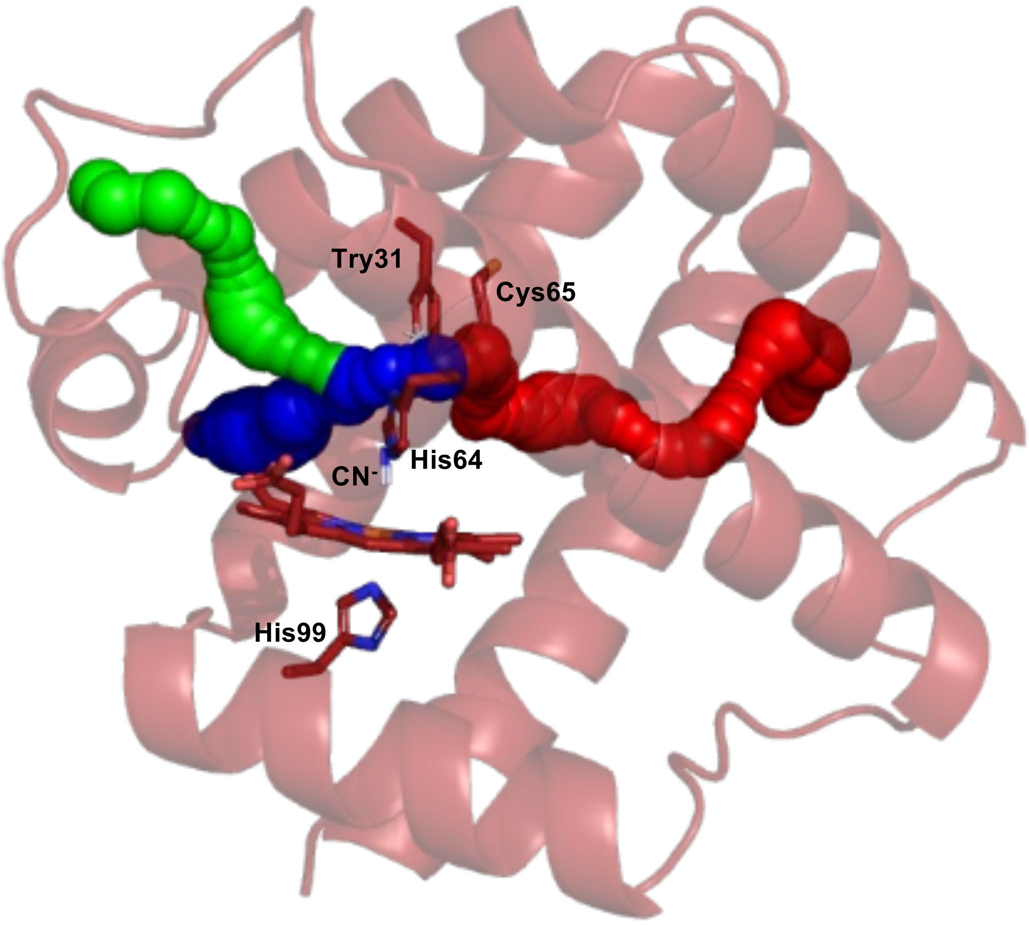

Fig. S9. The three tunnels (surface representation) identified in the crystal structure of Glb 2-1 (red, pdb 9R3P) by CAVER 3.0, through which O_2 could reach the heme cavity. The key residues and the cyano group are labeled and shown as sticks. Cys65 is within 3.0 Å distance from the three individual tunnels.
